# Mechanisms underlying the tissue-specific and regulated activity of the *Gnrhr* promoter in mammals

**DOI:** 10.3389/fendo.2012.00162

**Published:** 2012-12-13

**Authors:** Anne-Laure Schang, Bruno Quérat, Violaine Simon, Ghislaine Garrel, Christian Bleux, Raymond Counis, Joëlle Cohen-Tannoudji, Jean-Noël Laverrière

**Affiliations:** Physiologie de l’Axe Gonadotrope, Biologie Fonctionnelle et Adaptative, EAC CNRS 4413, Sorbonne Paris Cité, Université Paris Diderot-Paris 7Paris, France

**Keywords:** GnRH receptor, promoter regions, transcription, gonadotrope cell lines, steroidogenic factor 1, homeodomain proteins, transgenic mice

## Abstract

The GnRH receptor (GnRHR) plays a central role in the development and maintenance of reproductive function in mammals. Following stimulation by GnRH originating from the hypothalamus, GnRHR triggers multiple signaling events that ultimately stimulate the synthesis and the periodic release of the gonadotropins, luteinizing-stimulating hormone (LH) and follicle-stimulating hormones (FSH) which, in turn, regulate gonadal functions including steroidogenesis and gametogenesis. The concentration of GnRHR at the cell surface is essential for the amplitude and the specificity of gonadotrope responsiveness. The number of GnRHR is submitted to strong regulatory control during pituitary development, estrous cycle, pregnancy, lactation, or after gonadectomy. These modulations take place, at least in part, at the transcriptional level. To analyze this facet of the reproductive function, the 5′ regulatory sequences of the gene encoding the GnRHR have been isolated and characterized through *in vitro* and *in vivo* approaches. This review summarizes results obtained with the mouse, rat, human, and ovine promoters either by transient transfection assays or by means of transgenic mice.

## INTRODUCTION

Two GnRH systems (GnRH decapeptides and specific receptors) originally existed in the common mammalian ancestor but a large number of extant species have lost either the second type GnRH or its receptor (Stewart et al., 2009). Humans possess GnRH-II but lack a functional type-II receptor whereas mouse and rats lack both of them. In this paper, we will be dealing with the only GnRH system that has been proved to be involved in the reproductive hormonal cascade and that was retained in either humans or the two rodent models. The pituitary GnRH receptor (GnRHR) plays a central role in mammalian reproductive function since it establishes a unique molecular link between its ligand, the decapeptide GnRH originating from the hypothalamus, and the gonadotrope cells in the anterior pituitary. The hypothalamic GnRH is released into the portal hypophyseal vasculature in a periodic manner through a pulse generator that, likely by means of both gap junctions and voltage-gated calcium channels, coordinates the activity of individual neurons diffusely distributed in the hypothalamus (Vazquez-Martinez et al., 2001). Within the anterior pituitary, GnRH binds to specific high-affinity receptors present at the surface of the gonadotrope cells and induces increase in the synthesis and pulsatile release of the gonadotropins, luteinizing and follicle-stimulating hormones (LH and FSH). The pituitary hormones, which are composed of a common α- and distinct β-subunits, then enter the systemic circulation and, *via* specific receptors, modulate gonadal functions including gametogenesis, steroidogenesis, and ovulation. The amplitude and frequency of GnRH pulses relayed by the GnRHR appear critical in the development of the reproductive function, in the onset of puberty and throughout the menstrual or estrous cycle.

### INTRACELLULAR SIGNALING AND GnRHR-MEDIATED EFFECTS

The activation of GnRHR may trigger several intracellular signaling pathways in gonadotrope cells depending on the cellular context. Two mouse gonadotrope tumor-derived cell lines expressing the GnRHR, αT3−1 and LβT2 cell lines, have been used as homogenous cell models to study gonadotrope function (Windle, et al., 1990; Alarid et al., 1996; Thomas et al., 1996; Turgeon et al., 1996). In both cell lines, GnRH activates the protein kinase C (PKC)-dependent signaling pathway through coupling to G proteins of the Gq/G11 family (see review in Anderson, 1996). In primary culture of rat pituitary cells under sustained GnRH stimulation, a cAMP/PKA pathway is preferentially recruited (Garrel et al., 2010). In the gonadotrope-derived LβT2 cell line, sustained stimulation of GnRHR activates the cAMP signaling pathway through PKC δ and ε (Larivière et al., 2007). Also, in these cells, GnRHR signaling has been shown to further involve the three mitogen-activator protein kinases (MAPK) subfamilies (Liu et al., 2002). In non-gonadotrope cells like insect or rat lactosomatotrope cells stably transfected with GnRHR, the signaling mechanisms may involve the PKA pathway *via* Gs or Gi (Stanislaus et al., 1996; Delahaye et al., 1997; see review in Kraus et al., 2001). GnRHR activation generates several intracellular processes in the gonadotropes leading to enhanced transcription of the common α and specific LH and FSHβ subunit genes and secretion of these gonadotropins. Among these processes have been described activation of the neuronal nitric oxide synthase, induction of *cfos* expression, increase in the early growth response protein 1, increase in mRNA for annexin 5 and activation of translation and phosphorylation of the pituitary adenylate cyclase activating polypeptide (PACAP) type 1 receptor (see review in Garrel et al., 1995, 1997, 1998; Lozach et al., 1998; Brown and McNeilly, 1999; Sosnowski et al., 2000; Bachir et al., 2001, 2003; Duan et al., 2002; Kawaminami et al., 2002; Liu et al., 2002; Larivière et al., 2008). A global profile of genes regulated by GnRH in the LβT2 gonadotrope cell line was established through microarray analysis showing that more than 200 genes are either up- or down-regulated after GnRH agonist treatment (Kakar et al., 2003). The amplitude of these events is strictly dependent on the number of GnRHR molecules at the surface of the pituitary gonadotropes that is itself dependent, at least in part, on the transcriptional level of GnRHR gene (*Gnrhr*).

### GENE STRUCTURE AND PATHOLOGIES ASSOCIATED WITH MUTATIONS AFFECTING THE HUMAN GENE

**FIGURE 1:**
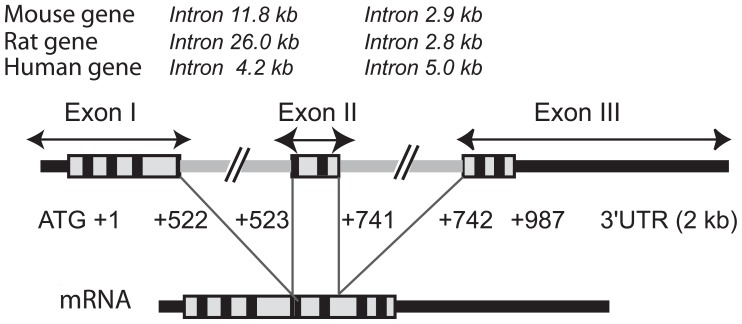
**Structure of the GnRH receptor gene**. The GnRH receptor gene is 15–31 kb in size depending on the species. This is primarily due to differences in intron sizes which are indicated at the top of the figure for mouse, rat, and human species. The coding sequence is boxed with the trans-membrane domains specified in black. The 5′ and 3′ untranslated regions (UTR) are designated by thick black lines whereas the two introns are specified by thick gray lines. The mature mRNA resulting from usual intron splicing is illustrated below the gene. Sizes of translated sequences included in the human mature transcript are indicated (987 bp corresponding to 328 codons excluding the stop codon). Rat and mouse translated sequences are 327 amino acids long, the exon II being three bases shorter (+523/+738).

The cloning of its encoding cDNA, firstly in the mouse and subsequently in other mammalian species, revealed that GnRHR is 325–328 amino acid long (327 in mouse and rat, 328 in human) and confirmed that this receptor belongs to the serpentine family of transmembrane G protein-coupled receptors (review in Norwitz et al., 1999a and references therein). The cDNA probes have subsequently been used to characterize the gene and its chromosomal localization in various mammalian species. *Gnrhr* is composed of three exons and two introns and is approximately 15–31 kb in size depending on the species (**Figure 1**). It is localized to chromosome 4q21.2 in human, chromosome 4, 5, 6, or 8 in bovine, murine, ovine, and porcine species, respectively (Fan et al., 1994; Kaiser et al., 1994; Morrison et al., 1994; Kakar and Neill, 1995; Kottler et al., 1995; Leung et al., 1995; Montgomery et al., 1995; Connor et al., 1999; Jiang et al., 2001). Cloning of the human *GNRHR* cDNA has led to the identification of mutations in the coding sequence that are associated with variable clinical features ranging from partial to complete hypogonadotropic hypogonadism (Achermann et al., 2001; de Roux and Milgrom, 2001). Two separate groups initially reported loss-of-function mutations in the *GNRHR* gene in patients with isolated hypogonadotropic hypogonadism (de Roux et al., 1997; Layman et al., 1998). A natural knockout of the human *GNRHR* resulting from aberrant splicing that eliminates exon 2 and creates a frame shift in the coding sequence was reported (Silveira et al., 2002). The woman affected by this homozygous mutation presented with primary amenorrhea, absence of gonadotropin pulsatility and did not respond to exogenous pulsatile or acute GnRH administration. To date, at least 20 additional mutations in *GNRHR *have been identified in patients with sporadic and familial isolated hypogonadotropic hypogonadism (Chevrier et al., 2011; Noel and Kaiser, 2011). These data again lay emphasis on the predominant role of *GNRHR* in the development of reproductive function in humans.

### TISSUE- AND CELL-SPECIFIC EXPRESSION OF THE GnRHR

The isolation of the cDNA also provided the basic tools for initiating studies on the regulation of *Gnrhr* expression by *in situ* hybridization, northern blotting, and semi quantitative RT-PCR. The presence of transcripts for the GnRHRs have notably been observed in brain regions previously shown to bind radiolabeled GnRH, thus confirming that *Gnrhr* is expressed in non-pituitary tissues, notably in the hippocampus and hypothalamus (see review in Jennes et al., 1997). *Gnrhr* is also expressed in granulosa cells of atretic follicles in the ovary, in Leydig cells in the testis, in prostate, breast, and placenta (Stojilkovic et al., 1994). In the pituitary, the expression of *Gnrhr* is restricted to the gonadotrope cells despite the common origin of the six different secreting cell types that composed the anterior pituitary (Scully and Rosenfeld, 2002). These diverse and discrete sites of expression raise the question of the mechanisms that determine the tissue-specific expression of *Gnrhr*. In addition, *Gnrhr* is regulated by extracellular signals, the first of them being GnRH itself. Endocrine factors such as estradiol (E_2_), progesterone, or testosterone also regulate the levels of *Gnrhr* mRNA (Stojilkovic et al., 1994). Altogether, these data led several groups to investigate the mechanisms that underlie the tissue-specific expression and regulation of *Gnrhr* (see review in Hapgood et al., 2005).

To this aim, the 5′ regulatory sequences of the rat, mouse, and human receptor genes have been isolated and characterized essentially by transient transfection assays and gel-shift analyses. A limited number of studies have also been conducted *in vivo* through the elaboration of transgenic mouse lines harboring a reporter gene under the control of the mouse, rat, or ovine promoter. This review focuses on the present state of knowledge related to the cell-specific and regulated activity of the promoter of the *Gnrhr in vitro* and *in vivo*.

## *IN VITRO* ANALYSIS OF THE MOUSE, RAT, AND HUMAN PROMOTERS

The tissue-specific and regulated activity of the rodent promoters have been evaluated using three main cellular models; the αT3−1 and LβT2 mouse gonadotrope-derived cell lines that are commonly used for studying the expression of marker genes of the gonadotrope lineage (Windle, et al., 1990; Alarid et al., 1996; Thomas et al., 1996; Turgeon et al., 1996) and a rat cell line of lactosomatotrope origin, the GGH(3) cells which were obtained by stable transfection with GnRHR of the tumor-derived GH3 cell line (Janovick et al., 1994; Kaiser et al., 1994; Stanislaus et al., 1994). αT3−1 cells express early marker genes of the developing gonadotrope in mouse, the gene encoding the common α-subunit of the three pituitary glycoprotein hormones (*Cga*) detected at embryonic day 11.5 (E11.5), *Gnrhr* detected at E13.5 and steroidogenic factor 1 (*Sf1*, formally nuclear receptor subfamily 5, member 1, NR5A1) detected at E14.5. The LβT2 cell line further expresses later marker genes, namely those encoding the β-subunits of LH (*Lhb*), from E16.5 onward, and FSH (*Fshb*), from E17.5. These data together with the way by which these cell lines were generated – by targeted oncogenesis using the promoter of *Cga* and *Lhb*, respectively – strongly suggest that the αT3−1 cell line may be representative of the gonadotrope lineage at early developmental stages, between E14 and E16.5. In contrast, the LβT2 cell line would be derived from cells at later stage, beyond E17.5 (Ingraham et al., 1994; Japón et al., 1994; Granger et al., 2004). It has to be emphasized that GGH3 cells express different tissue-specific transcription factors such as pituitary-specific transcription factor 1 (PIT1 formally POU domain class 1 transcription factor 1, POU1F1) that directs the expression of *Prl*, *Gh*, and *Tshb *(Bodner et al., 1988; Ingraham et al., 1988; Li et al., 1990; Ngô et al., 1995). Also, gonadotrope and lactosomatotrope cells likely express ubiquitous transcription factors at different levels. Moreover, as already mentioned above, the GnRHR seems to be coupled to slightly different signal transduction pathways in these cell lines. Consequently, the results obtained with these cellular models may reflect these major differences.

### ELEMENTS INVOLVED IN THE GONADOTROPE-SPECIFIC ACTIVITY OF THE MOUSE AND RAT *Gnrhr*

#### The mouse promoter (Figure 2A)

**FIGURE 2:**
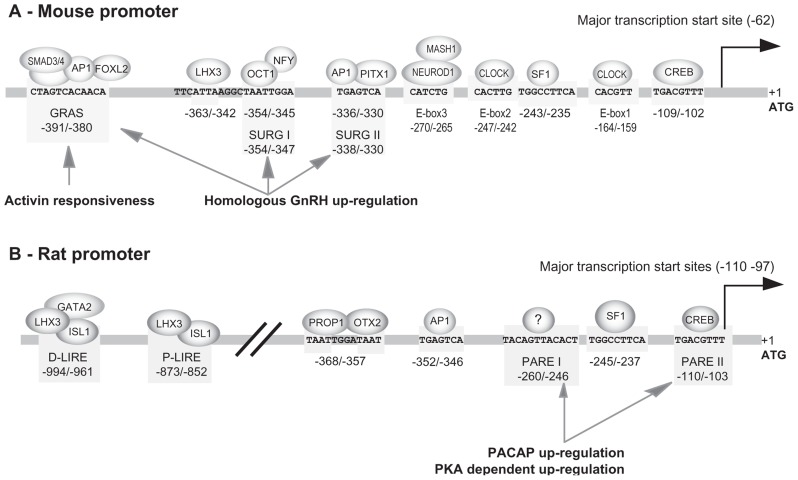
**Response elements identified within the mouse and rat promoters**. The schema summarizes the functional elements characterized on mouse **(A)** and rat **(B)** promoters. These elements are involved in tissue-specific expression in gonadotrope cell lines, some of them being also involved in activin-, GnRH-, and PACAP-dependent regulation. Note that response elements directing gonadotrope-specific expression of the rat promoter are located within 1000 bp 5′ upstream of the ATG codon whereas those of the mouse promoter are confined within the most proximal 400 bp. The numbering is relative to the translation start codon, the adenine being considered at position +1. This numbering may therefore be different from that used in the original publications.

***SF1, AP1, and GRAS elements***. The 1.2 kb promoter of the mouse *Gnrhr *was the first to be isolated and characterized in 1994 by Albarracin et al. (1994) Shortly after, a 1.9 kb mouse promoter was further isolated and functionally characterized (Clay et al., 1995). Both studies allowed identification of multiple transcription start sites within the first 100 bp immediately upstream from the ATG codon, the major one being located 62 nucleotides from the translation start codon. This transcription start site was referred to as position +1 in papers dealing with the mouse promoter. However, owing to the presence of multiple transcription start sites in GnRHR promoters and in order to facilitate the comparison between promoters of different species, we chose to use the translation start site as the position +1 in this review. Consequently, numbering in this review differs by +63 nucleotides from that currently used for the mouse promoter. This core promoter domain does not exhibit any typical CAAT or TATA boxes close to the transcription start sites. Both studies also demonstrated that the highest levels of promoter activity after transient transfection were obtained in mouse αT3−1 gonadotrope-derived cells. In rat lactosomatotrope cell line (GH3) or human JEG−3 placental cells, the activity of the mouse promoter was considerably weaker and represented only 17 and 5% of that observed in αT3−1 cells, respectively. Progressively, it appeared that all the positive regulatory elements involved in the tissue-specific activity of the mouse promoter in gonadotrope cells were confined into the 500 bp proximal region (Duval et al., 1997a,b). Three distinct motifs making up a tripartite basal enhancer are essentially required: a SF1 (NR5A1) response element (−243 5′-TGGCCTTCA−3′ −235), a canonical activating protein 1 (AP1) motif (−336 5′-TGAGTCA−3′ −330), and a novel element termed GnRHR activating sequence (GRAS; −391 5′-CTAGTCACAACA−3′ −380; **Figure 2**). Mutation of all three elements fully abrogates promoter activity. The identity of SF1 was further corroborated by gel-shift and antibody super-shift assays. Using similar approaches, the AP1 complex has been found to involve factors likely belonging to the FOS and JUN families. The nature of the factors that bind the GRAS element were also deciphered. They consist of SMAD3 (mothers against decapentaplegic homolog 3) which interacts with SMAD4 and bind to the 5′ end of the GRAS motif, AP1 that binds within the middle of the motif, and FOXL2, a member of the forkhead family, that interacts with the 3′ end of the GRAS motif. GRAS is thus a composite regulatory element whose functional activity is dependent on a multi-protein complex (Ellsworth et al., 2003a).

***Homeobox transcription factors***. Several homeobox factors further participate in the combinatorial code that directs gonadotrope-specific expression of the mouse *Gnrhr* promoter. The paired-like homeodomain transcription factor 1 (PITX1) belongs to the expanding family of bicoid-related vertebrate homeobox genes. It appears very early, being involved in the specification of the adenohypophyseal placode and its expression in the anterior pituitary persists afterward until adulthood (Drouin et al., 1998; Schlosser, 2006). PITX1 regulates different marker genes in the adult pituitary notably *Cga*, *Lhb*, *Fshb*, *Tshb*, *Gh*, *Prl*, *Pomc*, and *Gnrhr *(Drouin et al., 1998). PITX1 activates the *Gnrhr* promoter by interacting with c-JUN in the AP1 complex and by binding to several low affinity response elements scattered within the −370/−326 region of the mouse promoter that includes the AP1 response element (Jeong et al., 2004). Octamer-binding transcription factor 1 (OCT1, formally POU2F1) belongs to the other members of homeodomain transcription factors that regulate the *Gnrhr* promoter activity. This was demonstrated by several convergent approaches including targeted mutagenesis coupled with gel-shift assays and transient transfection in gonadotrope cell lines, chromatin immunoprecipitation (ChIP) assay, and short interfering RNA strategy (Kam et al., 2005). Together with the discovery of the OCT1 response element that contains a core TAAT motif at position −352/−349, a functional binding site for nuclear factor Y (NFY) was identified in the course of this study. It partially overlaps the OCT1 response element. Both transcription factors contribute to the tissue-specific activity of the mouse *Gnrhr* promoter. Several other TAAT motifs are present in the sequence of the mouse promoter. One of them, located at position −164/−159, mediates OCT1 trans-acting stimulation of *Gnrhr* promoter in addition to the distal OCT1 interacting motif (Lents et al., 2009). Additional homeodomain factors are involved in promoter activity. LHX3 that belongs to the LIM homeodomain (LIM-HD) proteins is expressed in the early stages of pituitary ontogenesis at E9.5 and its expression persists in the adulthood (Ericson et al., 1998). Similarly to PITX1, LHX3 is involved in the expression of several pituitary marker genes. An ATTA motif located at −360/−357 was found to mediate the mouse *Gnrhr* promoter responsiveness to LHX3. Also, the presence of LHX3 on the mouse promoter was demonstrated *in vivo *by ChIP assay (McGillivray et al., 2005). A complementary study showed that responsiveness to LHX3 was further dependent on a second TAAT motif located only 4 bp downstream (Cherrington et al., 2006). The integrity as well as the specific helical orientation of the two motifs are required for full LHX3 activity.

***E-box transcription factors***. Seven non-canonical E-boxes have been identified along the *Gnrhr* promoter sequence suggesting that basic/helix-loop-helix (b/HLH) transcription factors might be involved in tissue-specific promoter activity. b/HLH transcription factors (124 in numbers in mouse) have been classified into six groups based on their evolutionary relationship, protein structure characteristics, and sequence binding affinities (Li et al., 2006). Four of them, belonging to class A, B, and C, are suspected to modulate the activity of the mouse *Gnrhr *promoter. Indeed, mutation of each E-box in the mouse promoter resulted in full or severe decrease in promoter activity as measured by transient transfection assays using the αT3−1 gonadotrope cell line (Resuehr et al., 2007). One at least out of the three most proximal E-boxes (E-box 1, 2, and 3), binds the CLOCK (circadian locomotor output cycles kaput) transcription factor as demonstrated by ChIP assay. This strongly suggested that the mouse promoter is under the control of CLOCK/BMAL1 (brain and muscle arnt-like protein 1) heterodimer. In addition, transcripts encoding *Drosophila* homolog of period 1 (PER1), PER2, cryptochrome 1 (CRY1), and CRY2 were detected, signifying that dynamic inhibition by CRY/PER of CLOCK/BMAL1-directed gene transcription may take place in this gonadotrope cell line. Although the actors of the molecular loop essential for rhythmic timekeeping are all present, circadian rhythms of *Gnrhr* expression were not observed in cultured gonadotrope αT3−1 cells. Subsequent study considering E-box transcription factors has demonstrated the involvement of two other b/HLH transcription factors, neurogenic differentiation 1 (NEUROD1) and mammalian achaete-scute homolog 1 (MASH1), that both interact with E-box 3, suggesting that the CLOCK/BMAL1 heterodimer bind to either E-box 1 or 2 but not to E-box 3 (Cherrington et al., 2008). Interestingly, the αT3−1 gonadotrope cell line preferentially expresses NEUROD1 whereas LβT2 cells preferentially express MASH1. Consequently, *Gnrhr* E-box 3 binds NEUROD1 from αT3−1 cells, but binds MASH1 from LβT2 cells. This difference may be related to the differentiation state of these cell lines as mentioned above.

#### The rat promoter (Figure 2B)

**FIGURE 3:**
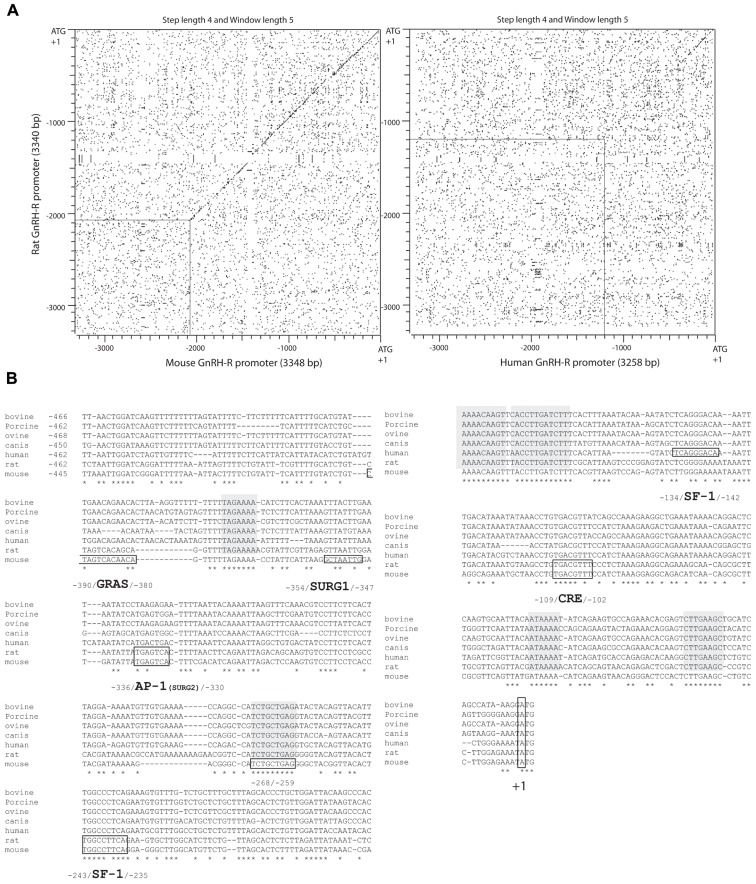
**Sequence comparison of *Gnrhr* promoters in different mammalian species**. **(A)** Dot matrix representation of pairwise alignment of rat versus mouse (left panel) or human (right panel) sequences. A strong sequence conservation between mouse and rat 5′ upstream flanking regions extends over 2 kb whereas that between rat (or mouse) and human sequences is limited to a proximal 0.5 kb region upstream of the ATG codon. Relaxed sequence identity between rat and human sequences is further observed between 0.5 and 1.2 kb upstream of the ATG codon. **(B)** Multiple sequence alignment of mammalian *Gnrhr* proximal promoters. Blocks of full identity are highlighted in gray. Some motifs of interest are boxed in the sequences of the species where they have been characterized (e.g., SURG1 in the mouse, AP1 in rat and mouse). Note that the SF1 response element characterized in the rat and mouse promoter sequences (−243/−235) is particularly well conserved among species contrasting with the human SF1 response element (−134/−142). The latter appears to be shared by bovine, porcine, ovine and canis species but not by rat and mouse.

***SF1, CRE, SAP, and AP1 elements in the proximal active domain***. The 5′ flanking sequence of the rat promoter has been successively isolated by Reinhart et al. (1997) and by Pincas et al. (1998) in our laboratory. Comparative analyses reveal 82–84% sequence identity with the corresponding 1.9 and 1.2 kb mouse promoter region. Sequence homology is well illustrated by dot matrix pairwise alignment of rat versus mouse sequence (**Figure 3A**). The analysis further reveals full lack of homology beyond 2.1 kb, suggesting that master regulatory domains in rodents are constrained within 2 kb upstream of the ATG codon. Furthermore, the transcriptional start sites appear similarly clustered within the 110 bp proximal region. A first set of major sites is located between −110 and −103, depending on the authors and a second set of both major and minor sites is located around position −30. The rat promoter was proved to be strongly active in αT3−1 and LβT2 gonadotrope-derived cells as well as in GT1−7 hypothalamic-derived cell line. Within the proximal domain, two elements that are identical to the AP1 (−352/−346) and SF1 (−245/−237) response elements of the mouse promoter are required to mediate gonadotrope-specific activity (Pincas et al., 2001a). However, despite these remarkable similarities, the rat promoter displays functional characteristics that distinguish it from the mouse promoter. Two additional response elements are required in the proximal active domain for full cell-specific activity (Granger et al., 2006) a CRE (cAMP response element) element (−110 TGACGTTT −103) that binds CRE binding protein (CREB) with a lower affinity than the consensus TGACGTCA sequence (Pincas et al., 2001b) and an element located at −252/−245 that binds a yet unidentified factor referred to as SAP for SF1 adjacent protein. Despite its immediate proximity with the SF1 response element, the SAP element acts independently of SF1. Furthermore, a GRAS-like element is present in the rat promoter but was proved inactive despite the close sequence identity with its mouse counterpart (Pincas et al., 2001a; Cherrington et al., 2005).

***Homeodomain factor response elements in the distal and proximal regulatory domains***. The distribution of response elements along the promoter constitutes the most important difference between the rat and mouse species. In contrast with the mouse promoter where all known response elements are clustered within the 500 bp proximal region, an additional regulatory region containing a bipartite enhancer that we named *Gnrhr*-specific enhancer (GnSE) lies on a more distal part of the rat promoter (−1135/−753). Two major response elements located at positions −994/−960 and −871/−862 are responsible for GnSE action (Pincas et al., 2001a; Granger et al., 2006). Interestingly, GnSE activity is dependent on both cellular and promoter contexts. Maximal activity required the presence of the SF1 response element that lies on the proximal promoter domain, 600 bp downstream. Thus, the GnSE belongs to a class of promoter-specific enhancers, capable of acting at a distance as classical enhancers do, but requiring a specific context, in our case, a SF1 element containing promoter. Regarding the proximal GnSE element (−871/−862), it was found to bind the pair of LIM-HD proteins LHX3 and ISL1 (Granger et al., 2006). This was demonstrated by several convergent approaches, notably using overexpression of the LIM-HD proteins and a LHX3 dominant negative in co-transfection assays, gel-shift and super-shift assays, and DNA affinity chromatography. This element displays high sequence identity in the antisense orientation with the −363/−342 region of the mouse promoter that was itself shown to interact with LHX3 (McGillivray et al., 2005; Cherrington et al., 2006). Using antibodies against LHX3 and ISL1 in ChIP assays, we were able to show that the mouse sequence did recruit ISL1 in addition to LHX3 (Schang et al., 2012b) as already demonstrated by McGillivray et al. (2005). Nevertheless, deletion of the LIM-HD response element in the rat promoter does not fully abolish the trans-acting effect of the ISL1/LHX3 pair. This led us to identify the upstream element of the GnSE as a second LIM-HD response element. It displays similar properties and specificity as the first characterized one (Schang et al., 2012b). These elements were then referred to as proximal and distal LIM-HD response elements, P-LIRE and D-LIRE, respectively. Finally, recent work from our laboratory has permitted to the identification of a prophet of PIT1 (PROP1)/orthodenticle drosophila homolog 2 (OTX2) response element in the proximal regulatory domain of the rat promoter, located at −368/−357. Interestingly, OTX2 (or OTX1) stimulates promoter activity in synergy with PROP1 by interacting with this element in gonadotrope-derived cell lines. In contrast, in non-gonadotrope cells, notably in the neutral Chinese hamster ovary (CHO) cell line, OTX2 acts synergistically with CREB through another OTX2 response element involving a ATTA core motif located at position −163/−160, close to the CRE element (Schang et al., 2012a).

#### Negative regulatory elements in the proximal part of the mouse promoter

As underlined above, the GH3 lactosomatotrope-derived cell line expresses the mouse promoter at a low but detectable level. The stably transfected GGH(3) cell, first designed in order to evaluate GnRH action, was thereafter used to identify the elements that allow constitutive expression in this non-gonadotrope, SF1-lacking cell line (Maya-Nunez and Conn, 1999). A CRE element (−109 5′-TGACGTTT−3′ −102) contributed to basal promoter activity, suggesting that constitutive activation of the PKA-dependent signaling pathway may also participate in cell-specific activity in pituitary gonadotropes as well as in other GnRHR expressing tissues. Importantly, several negative regulatory regions were identified (−213/−207; −268/−259; −354/−342) that may serve to repress the activity of the *Gnrhr* in non-gonadotrope pituitary cells. Among them the −268/−259 (5′-TCTGCTGA−3′) region is strictly conserved among bovine, porcine, ovine, canis, human, rat, and mouse sequences (**Figure 3B**). However, the inhibitory effects of these sequences are modest and cannot account for the whole lack of *Gnrhr* expression in non-gonadotrope pituitary cells. Furthermore, they partially overlap regulatory regions characterized as positive in a gonadotrope cell context.

### RESPONSE ELEMENTS INVOLVED IN TISSUE-SPECIFIC MOUSE AND RAT PROMOTER ACTIVITY ARE ALSO INVOLVED IN REGULATION BY EXTRA-CELLULAR SIGNALS

It is often noticed that tissue-specific transcription factors participate either directly or as co-regulators in the modulation of promoter activity by extra-cellular signals. This is also true for the rodent *Gnrhr* since, as we shall see in more detail below, SF1 is involved in PACAP- and PKA-regulated activity, AP1 in the homologous up-regulation by GnRH, and GRAS in the up-regulation by activin.

#### GnRH homologous up-regulation in gonadotrope-derived cells (Figure 4)

**FIGURE 4:**
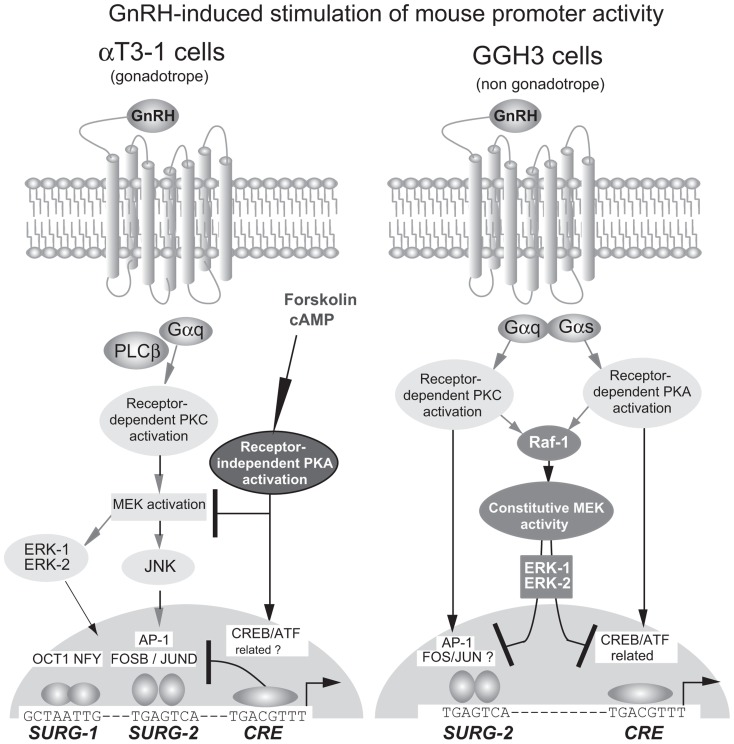
**Signaling pathways in the homologous up-regulation of the mouse GnRHR promoter by GnRH in gonadotrope and non-gonadotrope cells**. In αT3−1 cells, GnRH-induced activation of the PKC-dependent pathway stimulates promoter activity through the MAP kinase signaling pathway, involving ERK1 and ERK2. Receptor-independent activation of the PKA-dependent pathway inhibits GnRH-stimulated promoter activity. In GGH3 cells, GnRH induces the simultaneous activation of the PKC and PKA-dependent pathways for stimulating promoter activity. Constitutive activation of the MAP kinase signaling pathway, involving ERK1 and ERK2, inhibits basal and GnRH-stimulated promoter activity. The three elements, SURG−1, SURG−2, and CRE therefore participate in GnRH up-regulation in both αT3−1 and GGH3 cells. In αT3−1 cells, the positive involvement of SURG−1 and SURG−2 have been demonstrated whereas that of the CRE element is only suspected to be inhibitory. In GGH3 cells, the CRE element is required for promoter stimulation and appears as the ultimate target of the PKA-dependent pathway. AP1, also referred to as SURG−2 is only potentially involved essentially because GnRH also activates the PKC-dependent signaling pathway in these cells.

Two independent studies using transient transfection in αT3−1 cells demonstrated a time- and dose-dependent increase in the activity of the mouse *Gnrhr* promoter under GnRH treatment (Norwitz et al., 1999b; White et al., 1999). By using 3′ and 5′ deletion analyses combined with functional transfection studies, White et al. (1999) showed that a 54 bp fragment extending from −370 to −326 and thus including the AP1 element was sufficient to mediate GnRH response when placed upstream of a minimal heterologous promoter. Further analysis by Norwitz et al. (1999a) using linker scanning mutagenesis led to identifying two sequences of 8 bp, SURG−1 (sequence underlying responsiveness to GnRH−1; −354/−347) and SURG−2 (−338/−331) that are necessary for full GnRH responsiveness (**Figure 3B**). The SURG−2 element corresponds to the AP1 element and is absolutely required since mutations within this sequence totally abolished GnRH-induced stimulation of promoter activity whereas mutations within the SURG−1 element (5′-GCTAATTG−3′) only attenuated the response. Based on targeted mutagenesis of each of the GRAS, AP1, and SF1 elements that compose the tripartite basal enhancer, the study by White et al. (1999) has led to a rather similar conclusion regarding the AP1 element involvement. Both studies, using antibodies directed against conserved protein domains of either the JUN or FOS families in electrophoretic mobility shift assays strongly suggest that an AP1/DNA complex mediates GnRH homologous up-regulation. The MAPK kinase (MEK1/2) inhibitor PD98059 abrogates both GnRH-induced stimulation of promoter activity and GnRH-induced phosphorylation of extracellular signal-regulated kinases (ERK) 1 and 2 (White et al., 1999) and of c-Jun N-terminal kinase (JNK; Ellsworth et al., 2003b). GnRH induction of mouse *Gnrhr *promoter activity may thus require functional activation of a PKC-dependent pathway that involves the ERK- or JNK-signaling cascade or both and ultimately converges to AP1. However, accurate analysis of the intracellular events induced by GnRH treatment of αT3−1 cells strongly suggested that the JNK-signaling cascade is predominantly involved (Ellsworth et al., 2003b). Accordingly, in pituitary-targeted ERK1/2 double-knockout mice, *Gnrhr* expression was not altered as compared with wild-type animals (Bliss et al., 2009), suggesting that ERK signaling is not primarily involved in gonadotrope-specific expression of the *Gnrhr*. Moreover, the activation of the JNK-signaling cascade induced post-translational modifications of FOSB and JUND that led to increased binding of both factors on the SURG−2, the AP1 response element of the *Gnrhr* promoter (Ellsworth et al., 2003b). More recently, the factors that interact with SURG−1 have been identified as OCT1 and NFY. They are not only involved in the tissue-specific expression as mentioned above but also in GnRH homologous up-regulation. ChIP assays demonstrated that both factors are recruited on the mouse promoter in a time-dependent manner after GnRH treatment (Kam et al., 2005). It is important to point out that, in the context of the 600 bp mouse promoter, the adenylate cyclase activator forskolin inhibits GnRH-induced stimulation, suggesting a cross-talk between these two pathways (White et al., 1999). However, in the context of the chimera promoter made up with the mouse −370/−326 bp promoter fragment linked to the minimal GH heterologous promoter, forskolin is inefficient even if the GnRH response is maintained. This may indicate that the cross-talk takes place at the promoter level and requires response element(s) that are present within the 600 bp mouse promoter fragment and absent from the −370/−326 bp promoter fragment, notably the CRE element located at −109/−102 bp.

#### GnRH homologous up-regulation in non-gonadotrope cells (Figure 4)

The GnRHR is known to be expressed in several extra-pituitary tissues, notably in some areas of the brain, as well as in gonads and placenta. In this regard, it is important to consider the modalities of expression and regulation of the *Gnrhr* in cells distinct from gonadotropes. The availability of the stably transfected GGH3 cell line has permitted evaluating GnRH action on the transfected mouse promoter in a non-gonadotrope context. Consistent with the data obtained with αT3−1 cells, the activity of the mouse promoter was significantly increased in GGH3 cells following GnRH treatment (Lin and Conn, 1998). Subsequent analysis suggested that GnRH action is likely mediated by both PKA- and PKC-dependent signaling pathways (Lin and Conn, 1998, 1999; Maya-Nunez and Conn, 1999, 2001). Accordingly, GnRH stimulation was partly prevented by the adenylate cyclase inhibitor SQ22536 while treatment with the PKC inhibitor GF109203X led to nearly complete blockade. These data are in accordance with other results obtained using cholera toxin or cAMP analogs as well as following treatment with phorbol ester (Lin and Conn, 1998, 1999). It is important to specify that all these treatments resulted in the stimulation of the MAPK pathway in GGH3 cells (Han and Conn, 1999). However, agents that inhibit the activation of the MAPK pathway such as the drug PD98059 or ERK1 and ERK2 mutants together increased both tissue-specific and GnRH-stimulated promoter activity. Conversely, transfection of vectors expressing agents that mimic the activation of the MAPK pathway such as Raf1 or wild-type ERK1 and ERK2 inhibited tissue-specific and GnRH-stimulated promoter activity. Altogether, these data indicate that activation of the MAPK pathway is inhibitory in the lactosomatotrope GGH3 cell line whereas it is stimulatory in gonadotropes. Furthermore, GnRH action in GGH3 cells resulted in two opposite effects on the activity of the mouse *Gnrhr *promoter: a negative effect through the activation of the MAPK pathway and a positive one through the activation of PKA- and PKC-dependent pathways. The CRE element (−109/−102) that is involved in tissue-specific activity seems to be also involved in the GnRH and PKA-dependent regulation. The stimulatory effect of GnRH was indeed partially abolished and that of cAMP analog fully abrogated by targeted mutations of the CRE element. It is then rather probable that GnRH action also occurs through the SURG2 element in GGH3 cells.

#### Activin up-regulation

Activin, a member of the transforming growth factor-β superfamily, has first been shown to enhance the GnRH-induced FSH and LH release and to enhance the rate of GnRHR synthesis in rat pituitary cells (Braden and Conn, 1992; Weiss et al., 1993). This led to question its potential action at the transcriptional level. Exogenous activin B treatment of αT3−1 cells was shown to increase *Gnrhr* promoter activity as demonstrated by nuclear run-off assays or after transient transfection of the 1.2 kb mouse promoter linked to the luciferase reporter gene (Fernandez-Vazquez et al., 1996). However, since αT3−1 cells and pituitary gonadotropes both produce activin B, responsiveness to activin has then been mainly monitored by follistatin-induced inhibition of activin action in subsequent analyses (Duval et al., 1999). Using transient transfection in αT3−1 cells, follistatin inhibition of promoter activity was demonstrated to be mediated through the GRAS element. Indeed, mutation of this motif abrogated follistatin inhibition whereas alteration of the SF1 or AP1 elements was minimally efficient. Consistent with these results, three copies of the GRAS element were sufficient to confer activin responsiveness to a minimal heterologous promoter. The GRAS motif also exhibits sequence similarities with the element (GTCTAGAC) that binds the receptor-activated SMAD2/SMAD3 and the common SMAD4 (Stopa et al., 2000), involved in activin signaling (Walton et al., 2012). The autocrine/paracrine action of activin also explains why the GRAS motif has been classified as a paracrine/autocrine cell-specific response element. Comparison with the rat promoter sequence that does not exhibit activin responsiveness led to the characterization of a second region involved in activin-mediated up-regulation of the mouse promoter, a region referred to as “downstream activin regulatory element” or DARE (Cherrington et al., 2005). This 18 bp-long region lies between −366 and −349 and overlaps LHX3, OCT1, and NFY response elements. However, none of these factors seems to be involved in activin responsiveness. The integrity of the two TAAT motifs identified in this sequence was mandatory for functional DARE. Furthermore, appropriate helical orientation of this region relatively to the GRAS element located 20 bp downstream is required suggesting that factors that bind to DARE interact with GRAS binding factors (SMAD, AP1, FOXL2; Cherrington et al., 2006).

Norwitz et al. (2002a,b) have investigated the potential synergy between GnRH and activin treatment on the mouse promoter activity. They found that activin was able to increase the stimulatory effect of GnRH by approximately twofold. Interestingly, deletion of the promoter region that contains the GRAS element as well as mutation that alters the 5′ part of the GRAS element abrogated the effect of activin on GnRH stimulation. They also showed that overexpression of SMAD4 together with SMAD2 or SMAD3 increased both tissue-specific promoter activity and GnRH homologous up-regulation. However, it remains to be established if mutation of the presumed Smad binding element (SBE box −393/−386) in the GRAS element may prevent the stimulatory effect induced by SMAD overexpression. Competition and super-shift experiments further indicated that a second factor, belonging to the AP1 family, may bind the 3′ part of the GRAS element, suggesting that JUN/FOS related factors and SMAD protein functionally interact.

#### PACAP up-regulation

Specific type 1 receptors for PACAP are present in gonadotrope cells of the anterior pituitary gland as well as in mouse gonadotrope-derived αT3−1 cells. By transient transfection in αT3−1 cells, Pincas et al. (2001b) provided evidence that PACAP stimulates rat *Gnrhr* promoter activity. The EC50 of this stimulation was compatible with PACAP activation of the cyclic AMP-dependent signaling pathway and, consistently, co-transfection of an expression vector expressing the PKA inhibitor caused reduction in PACAP – as well as cholera toxin –stimulated promoter activity. Deletion and mutational analyses indicated that PACAP activation necessitated a bipartite response element that consists of a first region (−272/−237) termed PACAP response element (PARE) I that includes the SF1-binding site and a second region (−136/−101) referred to as PARE II that contains the CRE (TGACGTTT). Gel-shift experiments revealed that SF1 and SAP together bind to PARE I while a protein immunologically related to the CREB interacted with PARE II. Altogether, these findings indicated that PACAP regulates the rat *Gnrhr* at the transcriptional level in αT3−1 cells. Interestingly, the mouse promoter was similarly proved to be regulated by PACAP (Sadie et al., 2003). However, the response elements were not identified, nor were those involved in the PKA-induced stimulation of the mouse promoter. Notably, the ability of the CRE located at −109/−102 (i.e., equivalent to the rat promoter one) to mediate the PKA-dependent stimulation of the mouse promoter was not assessed. Nevertheless, two other response elements were shown to modulate PKA responsiveness (Sadie et al., 2003), the SF1 response element and a newly identified response element located at −15/−7, close to the translation initiation site (ATG). Both elements were able to bind NUR77 (NR4A1) and SF1 and exerted opposite effects. Mutation of the element close to the ATG codon together with overexpression of SF1 dramatically amplified PKA-induced stimulation. In contrast, overexpression of NUR77 repressed PKA-dependent regulation, most probably through the −15/−7 element. PACAP has been shown to stimulate follistatin (*Fst*) gene expression which restrains activin signaling and thus represses *Fshb* and *Gnrhr* expression as well as other activin-responsive genes. Therefore, in mouse gonadotrope cells, the PACAP direct stimulatory effect on *Gnrhr *promoter may be weakened by the indirect inhibitory action of PACAP on activin-stimulated promoter activity. The rat promoter is not submitted to this down-regulation because both the GRAS element and the DARE region are inactive. Whether PACAP regulation also occurs *in vivo* and is a common trait in mammals remains to be elucidated however.

### FUNCTIONAL CHARACTERISTICS OF THE HUMAN *Gnrhr* PROMOTER *IN VITRO*

The human *Gnrhr *promoter has been independently isolated by two groups (Fan et al., 1995; Kakar, 1997). It displays several features that distinguish it from rodent promoters (Cheng and Leung, 2005). Firstly, a significant sequence identity with the above-described promoters does not extend beyond approximately −1200 bp from the ATG codon (**Figure 3A**). Secondly, in contrast to the rodent promoters, the transcription start sites are located far upstream of the ATG codon and disperse within two domains localized between −578 and −826 and between −1347 and −1751, indicating the existence of two core promoter regions (Fan et al., 1995; Kakar, 1997). A further difference with mouse and rat promoters is the presence of several TATA boxes well scattered among the transcription start sites, suggesting that transcription initiation occurs through different mechanisms in rodent and human.

Given that GnRHR is expressed in several tissues in addition to the pituitary gland, functional studies with the human promoter have been performed in non-pituitary cell lines, notably cells derived from placenta, endometrium, trophoblast, and ovary. This strategy has also been motivated by the absence of pituitary cell line of human origin. These studies led to the discovery of several positive and negative regulatory regions, the efficacy of which being strongly dependent on the considered cell line. They essentially confirmed the existence of distinct promoters within the 2 kb human 5′ flanking sequence.

#### The proximal core promoter is predominantly active in gonadotrope-derived cells (Figure 5A)

**FIGURE 5:**
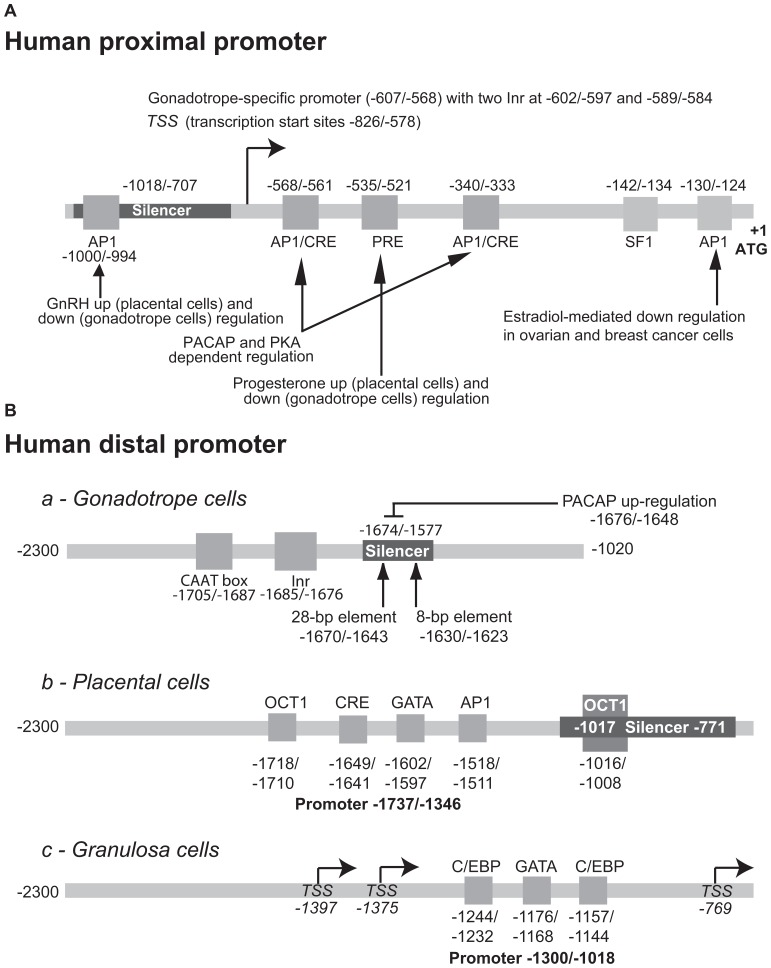
**Structure of the human GnRHR promoter**. The elements identified in the proximal **(A)** and distal **(B)** promoter of the human gene and involved either in the tissue-specific or regulated activity are indicated. To facilitate comparison with the rodent promoters, the first nucleotide of the ATG codon was considered as position +1. The numbering may therefore be different from that used in the original publications.

Following transfection in αT3−1 cells, it appeared that crucial elements are located in the close vicinity of the translation start codon because the −173/+1 deletion abolished promoter activity (Ngan et al., 1999; Kang et al., 2000). Targeted mutagenesis of a SF1 response element located at −142/−134 (5′-CAGGGACAA−3′) altered overall promoter strength suggesting that it is likely contributing to the gonadotrope-specific activity. The involvement of SF1 has been further evidenced by electrophoretic mobility shift and super-shift assays as well as by overexpression of SF1 (Ngan et al., 1999). However, silencer elements present in the middle part of the human promoter (−1018/−707) weaken promoter strength. Indeed, 5′ deletion to −707 increased tissue-specific activity in αT3−1 cells, the deleted promoter (−707/+1) being approximately threefold more active than the entire promoter (−2200/+1; Cheng and Leung, 2001). These data are in agreement with the existence of a proximal core promoter region (−816/−577) previously documented (Fan et al., 1995) and with the presence of a positive regulatory region (−771/−557) identified by 3′ deletion analysis (Kang et al., 2000). This also suggests that positive regulatory regions either overlap the core promoter region or are situated downstream from the transcription start sites as is the SF1 element cited above. Subsequent analysis by scanning mutations in αT3−1 cells has allowed precise delimitation of this proximal promoter that extends from −607 to −568. It contains two pyrimidine-rich initiator elements (−602/−597 and −589/−584) that bind similar proteins notably TFIID (Hoo et al., 2003). The distal promoter of the human gene is constitutively active in both gonadotrope and non-pituitary cell lines (**Figure 5B**).

The existence of a distal promoter active in non-pituitary cell lines has been strongly suggested in the early study by Kakar (1997). In this work, it is indeed shown that the −2300/−1020 human flanking sequence is highly active in human endometrial (HEC−1A) and breast tumor cell (MCF−7) lines. This upstream promoter was further characterized in either gonadotrope or non-pituitary cells by Ngan et al. (2000) and Cheng et al. (2001a), respectively. It appears to be less active in αT3−1 pituitary than in JEG−3 placental cells (Cheng et al., 2001a) and thus contrasts with the core proximal promoter that displays opposite characteristics. Also, the distal promoter seems to be more ubiquitous than the proximal promoter since it was active in gonadotrope, ovarian, endometrial, mammary, placental, and kidney cells. The in-depth analysis of the distal promoter in gonadotrope-derived αT3−1 cells (Ngan et al., 2000) and placental JEG−3 cells (Cheng et al., 2001a) reveals cell-specific differences.

In gonadotrope-derived cells, the active elements of the upstream promoter are located within a −1705/−1674 region and are not functional in the reverse orientation. They consist of a pyrimidine-rich initiator (−1685/−1676) and additional upstream motifs (−1705/−1687) that include a CCAAT box. Multiple proteins ranging from 40 to 54 K are capable of binding this upstream promoter. Interestingly, a downstream silencer element (−1674/−1577) prevents upstream promoter activity, at least in gonadotrope cells. This silencer is efficient only when positioned downstream of the promoter in either reverse or forward orientation. Two sequence-specific repressor motifs of 28 and 8 bp (−1670/−1643 and −1630/−1623) are essential for silencer activity as evidenced by linker-scanning mutagenesis and gel mobility shift assays (Ngan et al., 2001).

In JEG−3 and IEVT placental cell lines, a similar organization seems to occur, combining positive regulatory sequence and downstream silencer, however with different localization and distinct response elements (Cheng et al., 2001a). The placental cell-specific upstream promoter is located within a −1737/−1346 region and thus overlaps the gonadotrope cell-specific promoter. In JEG−3 cells, four motifs related to OCT1 (−1718/−1710), CRE (−1649/−1641), GATA (−1602/−1597), and AP1 (−1518/−1511) response elements are contributing to promoter activity. The characterization of these motifs has been further assessed by gel mobility shift and antibody super-shift assays. The downstream silencer is located between −1017 and −771 and abrogates upstream promoter activity in a manner similar to what is observed with the gonadotrope promoter. The silencer was further characterized in the context of the −1300/−1018 promoter described below. Under these conditions, it appeared that most of the silencing activity of this negative regulatory element resides in an octamer responsive sequence activated by OCT1 (Cheng et al., 2002b).

#### Distal promoter usages in granulosa and neuronal cell lines

A third distal promoter was characterized in human ovarian granulosa-luteal cells. It extends from −1300 to −1018 and exhibited the highest promoter activity in SVOG−4o and SVOG−4m immortalized granulosa-luteal cells (Cheng et al., 2002a). However, it was also active in placental, ovarian, and gonadotrope cells. Two CCAAT enhancer binding protein (C/EBP) located at −1244/−1232 and −1157/−1144 and one GATA response element located at −1176/−1168 were required for full promoter activity because simultaneous mutations of these three elements led to more than 75% decrease in promoter activity in granulosa cells. Interestingly, the same mutations induced minimal or no significant decrease in promoter activity in ovarian, placental, and gonadotrope cells suggesting that different response elements and different transcription factors are involved in these cell contexts.

Human GnRHR was further expressed in the human cerebellar medulloblastoma cell line TE−671. In these neuronal cells, promoter activity was dependent on an upstream region located between −2197 and −1018. Important *cis*-acting regulatory elements were identified at −1300/−1018 and −2197/−1900, as deletion of either region induced a dramatic decrease in promoter activity (Yeung et al., 2005).

### REGULATION OF HUMAN PROMOTER ACTIVITY BY EXTRACELLULAR SIGNALS (FIGURE 5)

#### Homologous up- and down-regulation by GnRH and the PKC-dependent pathway

Surprisingly, when compared to rodents, a 6–24 h-long treatment with 0.1 μM GnRH reduced activity of the 2.3 kb human *GnRHR *promoter by 31–71% in αT3−1 cells. This inhibitory effect was mimicked by phorbol ester and prevented by GnRH antagonists or specific PKC inhibitors (Cheng et al., 2000) strongly suggesting that GnRH action is mediated through receptor-dependent activation of the PKC pathway. Subsequent 5′ deletion analyses combined with site-directed mutagenesis revealed that an AP1 related element located at −1000/−994 (TTAGACA) is responsible for the homologous down-regulation of promoter activity by forming an AP1 complex involving the c-JUN and c-FOS proto-oncogenes. This AP1 element is unrelated to the AP1/CRE element involved in tissue-specific expression. It is worth stressing that similar GnRH treatment applied to placental JEG−3 or IEVT cell lines led to significant increases in promoter activity, contrasting again with data obtained in gonadotrope-derived cells (Cheng et al., 2000). The mechanisms underlying these positive and negative regulations remain to be elucidated, notably whether differential promoter usage (upstream versus proximal) may be related to such difference in GnRH regulation.

#### Up-regulation by PACAP and PKA-dependent pathway

The regulation of the human promoter by PACAP and the PKA-dependent activation pathway has been investigated in two different studies and led to the identification of distinct regulatory domains located within either the upstream or the proximal promoter, respectively (Cheng and Leung, 2001; Ngan et al., 2001). The former study (Ngan et al., 2001) indicated that both the gonadotrope cell-specific upstream promoter as well as the downstream silencer are involved in PACAP and PKA-dependent regulation. In support, PACAP treatment enhanced the formation of a complex with the 28 bp sequence-specific repressor motif (see above). Whether this may contribute to suppress silencer function and in turn to activate promoter remains to be demonstrated. The mechanisms underlying this positive regulation are not yet clearly identified. The latter study by Cheng and Leung (2001) led to the conclusion that PACAP and CREs reside within a 412 bp fragment (−577/−167) of the proximal promoter. Indeed, mutation of the (−568/−561 and −340/−333) AP1/CRE related motifs nearly abrogated the stimulation.

#### Up- and down-regulation by steroid hormones

Similarly to GnRH, progesterone treatment led to either inhibition or stimulation of promoter activity in gonadotrope versus placental cells, respectively (Cheng et al., 2001b). A progesterone binding site located at −535 to −521 has been shown to mediate, at least partially, both the inhibitory and stimulatory action of progesterone. Overexpression of the human progesterone receptor A and B isoforms increased the negative effect of progesterone in αT3−1 cells, whereas only B isoform mediated its positive action in JEG−3 placental cells. It is important to point out that the A isoform mediated inhibitory action in both cell lines, suggesting that the balance in the expression of A and B isoforms may be critical for GnRHR expression in the pituitary. The involvement of the progesterone receptor in binding the −535/−521 motif has been further substantiated by gel-shift and antibody super-shift assay.

In OVCAR3 ovarian carcinoma and MCF7 breast carcinoma cell lines, E_2_-induced repression of *Gnrhr* promoter activity through an E_2_ receptor α (ERα)-mediated mechanism (Cheng et al., 2003). This occurred in a dose- and time-dependent manner, the maximal effect being obtained at 10^−8^ M E_2_ after a 48 h-long treatment (80% reduction of promoter activity). Repression occurred *via* an AP1-like element located at position −130/−124. This element was able to bind c-FOS and c-JUN but not ERα as demonstrated by EMSA. DNA binding by ERα was not needed for the E_2_ repression of *Gnrhr *promoter activity. Further experiments strongly suggested that recruitment of AP1 and ERα to CREB binding protein (CBP) was mutually exclusive leading to inhibition of AP1-stimulated promoter activity by E_2_.

## *IN VIVO* ANALYSIS OF THE MOUSE, OVINE, AND RAT PROMOTERS IN TRANSGENIC MICE

Since *in vitro* studies of promoter functions often suffer from limitation of the cellular models used in transient transfection analyses, the next and complementary approach consists in analyzing promoter activity in an *in vivo* context through the generation of transgenic mice.

Several studies have been published that use this procedure. The initial study by McCue et al. (1997) demonstrated the homologous up-regulation of *Gnrhr* promoter activity by GnRH. A 1900-bp fragment corresponding the mouse *Gnrhr *promoter fused to the luciferase reporter gene has been microinjected in mouse embryos and mice harboring the transgene were selected. Analysis of luciferase activity in various tissues showed that the fusion gene was expressed essentially in the pituitary gland, to a 10-fold lower level in the brain and to a limited extend in the testis. These data are in agreement with those deduced from autoradiographic studies using ^125^I-labeled GnRH or *in situ* hybridization (Jennes et al., 1988, 1995; Leblanc et al., 1988; Ban et al., 1990; see review in Jennes et al., 1997). Surprisingly, the expression of the transgene was not detected in the ovary contrasting with a number of studies reporting the presence of GnRHR in this tissue, specifically in granulosa cells of atretic follicles. Using an antibody directed against GnRH, the authors further showed a decrease in transgene expression in the pituitary gland but not in the brain or testis. The GnRH antibody inhibitory action could be reversed by a GnRH agonist treatment. As expected from *in vitro* studies, the 1900-bp promoter contains essential GnRH response elements. However, E_2_ treatment of these transgenic mice did not alter the activity of the transgene. This diverges with a number of reports demonstrating that, *in vivo*, E_2_ increases GnRHR number and steady-state level of *Gnrhr* mRNA in the pituitary (Hamernik et al., 1995; Turzillo et al., 1995; Kirkpatrick et al., 1998).

The same group has thus reinvestigated this regulation with novel lines of transgenic mice harboring a fusion construct containing the ovine receptor gene promoter linked to the luciferase reporter gene (Duval et al., 2000). Using either a 9.1 or a 2.7 kb promoter, they first showed that the ovine transgene displayed the same tissue-specific expression as the mouse promoter. The ovine transgene was indeed consistently expressed in the pituitary, brain, and gonads across the three analyzed transgenic lines. Pituitary expression was higher in females than in males whereas ovarian expression was significantly lower than the testicular one. In agreement with results obtained with the mouse promoter, immunoneutralization of GnRH reduced pituitary-specific expression of the luciferase reporter gene in ovariectomized mice, expression that could be restored by E_2_ treatment. Similar results were observed in castrated, male transgenic mice. Finally, combined treatments with GnRH agonist and E_2_ increased pituitary expression of the luciferase fusion gene to an extent greater than the sum of each individual treatment, suggesting synergistic activation of the ovine promoter.

The third study involving transgenic mice was different in at least two facets. Firstly, in this case, the reporter gene was the simian virus 40 (SV40) large T antigen and, secondly, the mouse promoter was the shortest used since it was 1.2-kb long only (Albarracin et al., 1999). With this fusion construct, the resulting heterozygote transgenic female mice were infertile preventing the generation of homozygote animals. Interestingly, all transgenic animals developed large intracranial tumors by 4–5 months of age that were of pituitary origin. As evidenced by *in situ* hybridization, these tumors expressed the marker genes of the gonadotrope phenotype, i.e., GnRHR, glycoprotein hormone α-subunit as well as FSHβ and LHβ subunits. The large T antigen was also expressed in the pituitary tumors but neither in the gonads or in the brain. This may indicate that the 1.2-kb, as compared with the 1.9-kb promoter fragment, is not able to direct transgene expression to sites other than the pituitary, notably brain and gonads. Surprisingly, the expression of the SV40 large T antigen transgene driven by the GnRHR gene promoter results in female-specific infertility due to disruption of gonadotropin production and secretion even before tumor development (Jeong et al., 2009).

In our laboratory, the 3.2 kb rat promoter was placed upstream of the human placental alkaline phosphatase reporter gene (*ALPP*) and inserted into the mouse genome. The resulting transgenic mice expressed the ALPP exclusively in gonadotropes within the pituitary gland (Granger et al., 2004). Transgene expression was detected within the developing pituitary at E13.5 in some of the cells that also expressed the *Cga*. It showed that *Gnrhr *is the earliest marker of gonadotrope cell differentiation, before SF1 that is detected at E14.5. At E15.5, ALPP expression was localized in LHX3- and ISL1-immunoreactive positive cells in agreement with *in vitro* data showing that these LIM-HD proteins are involved in rat and mouse promoter activity. Importantly, the 3.2 kb promoter was also able to direct *ALPP* expression to several brain areas, in particular the hippocampus and the lateral septum, in agreement with previous data (Jennes et al., 1988, 1995; Leblanc et al., 1988; Ban et al., 1990; see review in Jennes et al., 1997). In contrast with the pituitary gland, transgene activity in the hippocampus was detected only after birth, increasing gradually until 14–20 days after birth and then remaining at constant level until 60 days after birth (Schang et al., 2011b). This occurred simultaneously in fibers extending from the hippocampus to the lateral septum, suggesting that GnRHR may be involved in post-natal maturation of the septo-hippocampal system. The same time-course was observed by measuring *Gnrhr* mRNA in rat hippocampus by quantitative RT-PCR, indicating that transgene expression directed by the rat promoter recapitulates rat endogenous *Gnrhr* expression in a mouse context. Further experiments using transient transfection of *Gnrhr* promoter luciferase fusion constructs in primary culture of rat hippocampus cell revealed that a RE1-silencing transcription factor (REST also known as NRSF)-like element located at −2.5 kb may be involved in the inhibition of *Gnrhr* gene expression before birth. Finally, GnRH treatment of rat hippocampus primary cell culture led to stimulation of several markers of neurogenesis such as EGR1, synaptophysin, and spinophilin. Using this transgenic mouse model, several additional sites of transgene expression were detected, notably in the oculomotor pathway (Schang et al., 2011a). Interestingly, transgene expression was also detected in two functionally and evolutionary related organs, the pineal gland and the retina. Again, the onset of transgene expression was specific to these tissues, being detected at E13.5 and E17.5 in the pineal gland and retina, respectively (Schang et al., 2012a). In the pineal gland, transgene expression persisted until adulthood whereas it strongly decreased in the retina. During development, transgene expression was the strongest in the neuroblast cell layer of the retina and less marked in the ganglion cell layer. In the pineal gland, transgene expression was constantly observed in approximately 50% of the cells whatever the developmental stage. Finally, transgene expression was strongly detected in the testes of transgenic mice in some Leydig cells (Schang et al., 2011a).

Recent studies involving functional genomic approaches have shown that regulatory domains may be located within the gene locus at several kilobases, either 5′ upstream or 3′ downstream as well as within intronic regions. This is well demonstrated particularly for genes coding for transcription factors, notably SF1 (Shima et al., 2012 and references therein), or genes encoding for factors involved in early developmental processes such as the LIM-HD proteins LHX3 and ISL1 (Kappen and Salbaum, 2009; Mullen et al., 2012). Conversely, other studies have shown that the most important genetic regulation may be contained within only 1 kb 5′ upstream of the transcription start site, including informations regarding epigenetic modifications such as pattern of DNA methylation (Lienert et al., 2011). Furthermore, species-specific informations would be encoded into DNA sequence and appears to prevail over species-specific cellular environment. A human chromosome introduced into a mouse cell line directs genetic and epigenetic modifications as it does in a human cell context and thus differently from its mouse counterpart (Wilson et al., 2008). Therefore, data obtained using transgenic mice as well as those described above must be considered bearing in mind the fact that all regulatory sequences are not necessarily contained within the 5′ upstream region of the gene only, even if relatively short 5′ upstream sequences may contain master regulatory informations. This could explain why the human *GNRHR* promoter responded negatively while mouse or rat promoters are stimulated by GnRH.

To overcome these limitations, a double knock-in mice model was recently developed. In this binary genetic approach, the Cre-recombinase was inserted into the *Gnrhr* locus whereas the YFP reporter gene, preceded by a floxed stop signal, was inserted into the ubiquitously expressed ROSA26 locus. The Cre-mediated excision of the stop signal led to a constitutive YFP expression in about 15% of the cells in the male anterior pituitary. Furthermore, the fluorescent signal was only colocalized with LHβ and/or FSHβ subunits as demonstrated by immunohistochemistry. These YFP mice have allowed single-cell analysis on living cells which have revealed unexpected heterogeneity in the resting properties of gonadotrope cells as well as in their secretory, electrophysiological, and calcium responses to GnRH (Wen et al., 2008).

## SF1, A PAN-SPECIES TRANSCRIPTION FACTOR CRITICAL FOR GONADOTROPE-SPECIFIC EXPRESSION

An obvious conclusion that emerges from this overview of the mechanisms involved in *Gnrhr* expression is the important role of the orphan nuclear receptor SF1. It is undoubtedly involved in the tissue-specific expression of the human, mouse, and rat *GnRHR*. It is also likely involved in the tissue-specific expression of the ovine gene since the promoter is strongly homologous to its human counterpart. SF1 is crucial not only for tissue-specific expression but also for the regulation by extracellular signals as evidenced by PACAP and PKA-dependent regulations of the rat promoter. This may be related to the other important functions of SF1 in the tissue-specific and regulated expression of *Cga* and *Lhb* genes, two other markers of the gonadotrope lineage. Consistent with these data, the SF1 knockout mice exhibit impaired expression of LHβ, FSHβ, and GnRHR in pituitary gonadotropes in addition to other major defects such as agenesis of the ventro-medial hypothalamus, complete adrenal and gonadal agenesis and male-to-female sex reversal with persistence of Müllerian structures in male (Ingraham et al., 1994; Ikeda et al., 1995; Luo et al., 1995). To bypass the influence of hypothalamic deficiencies inherent to this transgenic model, SF1 has been inactivated specifically in the anterior pituitary using the Cre-loxP system (Zhao et al., 2001). These pituitary-specific SF1 knockout mice are sterile and never mature sexually. Consistent with a direct role of SF1 at the pituitary level, FSH and LH are markedly decreased in the pituitary-specific SF1 knockout mice as well as are gonadotropin β-subunit and *Gnrhr *mRNAs as assessed by semi-quantitative RT-PCR.

The important role of SF1 has been further confirmed in humans by the discovery of a heterozygous missense mutation in the DNA binding domain of SF1 in a patient with complete sex reversal, testicular dysgenesis, and adrenal failure (Achermann et al., 1999). However, this patient displayed a relative preservation of gonadotrope function. This may be due either to its heterozygous genotype or to a relative autonomy of the gonadotrope function with respect to SF1. In agreement with the latest hypothesis, GnRH treatment can restore LH and FSH secretion in SF1 knockout mice (Ikeda et al., 1995), suggesting that SF1 is not required for the emergence and maintenance of gonadotrope function. Furthermore, some tissues known to express GnRHR such as amygdala and hippocampus are deprived of SF1 (Schang et al., 2011b). This suggests that other factor(s) than SF1 are needed for the tissue-specific transcriptional onset of the *Gnrhr*. To identify this (or these) factor(s), several parameters must be considered. From an ontogenetic point of view, the date of appearance of the *Gnrhr* transcripts must be correlated with the presence of the factor in the fetal pituitary or in fetal tissues that will express the GnRHR in the adult. For instance, binding sites for GnRH have been detected as early as 12–13 days of gestation in the rat developing pituitary and GnRHR mRNA at 14.5 and 15.5 days post-conception in testis and ovary from rat fetuses (Aubert et al., 1985; Jennes, 1990; Botté et al., 1998). This factor should also be able to activate the promoter of the *Gnrhr* in an SF1-independent manner in transient transfection assays.

## CONCLUSIONS AND PERSPECTIVES

An increasing number of studies on gene regulations and promoter activities aim to demonstrate the existence of specific regulatory units that bring together several response elements. From one tissue to another, the same elements may bind different factors resulting in tissue- or cell-specific modulations of gene expression. This ability to bind different factors is likely facilitated by sequence degeneracy of the response elements that lie within these regulatory units. Consequently, the degenerated response elements may be less efficient than their canonical counterparts when they are isolated from their promoter context. Efficiency is recovered in the original promoter context through synergistic interactions with other transcription factors belonging to the regulatory unit. Interestingly, this synergy may be attenuated if the wild-type degenerated element is artificially replaced by its canonical counterpart (Ito et al., 2000). In the *Gnrhr* promoters, several elements display such characteristics. In particular, the SF1 binding site and the proximal CRE in the rat and mouse promoter differ from the consensus sequence (TGACGTTT instead of TGACGTCA for the later). In gel-shift experiment, both the SF1 element and the CRE display an apparent weaker affinity for their cognate transcription factors than the SF1 binding site from the rat aromatase gene or the canonical CRE sequence (Pincas et al., 2001a,b). PACAP- and PKA-dependent regulations of the rat promoter are mediated by these two elements that may thus be considered as a cAMP regulatory unit (Roesler, 2000). The SURG−1 and SURG−2 elements establish another functional regulatory unit since they are both required for the GnRH up-regulation of the mouse promoter. The distal GnSE together with the proximal domain containing the SF1 binding site also form a regulatory unit for the tissue-specific activity of the rat promoter. Different regulatory units may also bind different combinations of factors depending on the tissue or the cell type that expresses the *Gnrhr*. We have recently demonstrated that OTX2 interacted with two distinct partners depending on the cell context (Schang et al., 2012a). In a gonadotrope cell context, OTX2 interacted with PROP1 on two adjacent response elements located between −388 and −357, each of them encompassing a core TAAT motif. In a neutral cell context, OTX2 acted in synergy with CREB and formed a regulatory unit that involved CRE, an AP1 response element together with a new OTX2 binding site containing a TAAT core motif located at −163/−160. This motif is thus located 50 bp upstream of the CRE and 180 bp downstream of the AP1 element establishing a regulatory unit extending over 200 bp within the proximal promoter, markedly larger than the PROP1/OTX2 regulatory unit (Schang et al., 2012a). Whether such difference in size of these regulatory units as well as the other cited above is functionally relevant remains to be determined. In this respect, an important point that must be considered is the chromatin structure that depends on epigenetic modifications. At least, two histone H3 modifications are predominantly involved in transcriptional regulation: tri-methylation of Lys 4 of histone H3 (H3K4me3) and tri-methylation of Lys 27 of histone H3 (H3K27me3; see review in Greer and Shi, 2012). They are linked to gene activity and gene silencing, respectively. H3K4me3 is catalyzed by trithorax group (trxG) proteins, whereas H3K27me3 is catalyzed by polycomb-group (PcG) proteins (Schuettengruber et al., 2007; Ku et al., 2008). PcG proteins reside in two main complexes, termed Polycomb repressive complexes 1 and 2 (PRC1 and PRC2). PRC2 catalyzes H3K27me3 and components of PRC1 are recruited to the modified histone leading to ubiquitinylation of histone H2A at lysine 119 which in turn prohibits RNA polymerase II elongation (Simon and Kingston, 2009). This results in stable transcriptional repression. Analysis of H3K4me3 and H3K27me3 modifications by ChIP assay showed that in gonadotrope cell lines, the *Gnrhr* promoter preferentially displayed the active mark whereas in lactosomatotrope or corticotrope cell lines, it displayed preferentially the repressive mark (unpublished data). This is well correlated with *Gnrhr* mRNA levels that are not detected in lactosomatotrope and corticotrope cell lines. Constitutive *Gnrhr* transcription is thus likely controlled by mechanisms involving trxG proteins in gonadotrope cells whereas in other pituitary cells, PcG proteins stably repress *Gnrhr* expression. In mammals, composition of trxG and PcG protein complexes displays broad diversity depending on the considered family of genes. A critical point would be to determine what are the main actors involved in histone methyltransferase and demethylase activity regarding the *Gnrhr*. Furthermore, PcG complexes are known to be clustered into subnuclear structures that look like nuclear foci termed polycomb bodies (Pirrotta and Li, 2012). Conversely, active genes are preferentially located in different subnuclear structures referred to as transcription factories that contain active RNA polymerase II or interchromatin granule clusters (also termed nuclear speckles) that contain histone-modifying enzymes, transcription elongation factors, and splicing machinery (Meldi and Brickner, 2011; Spector and Lamond, 2011). In the developing pituitary gland, lineage specification likely requires relocation of marker genes like the *Gnrhr* into these subnuclear structures (Yang et al., 2011). The elucidation of the mechanisms underlying this reorganization would permit to shed new light on *Gnrhr* expression, on gonadotrope cell lineage specification and differentiation, and more generally on organ development.

## Conflict of Interest Statement

The authors declare that the research was conducted in the absence of any commercial or financial relationships that could be construed as a potential conflict of interest.

## References

[B1] Achermann J. C. Ito M. Ito M. Hindmarsh P. C. Jameson J. L. (1999). A mutation in the gene encoding steroidogenic factor-1 causes XY sex reversal and adrenal failure in humans. *Nat. Genet.* 22 125–126 10369247 10.1038/9629

[B2] Achermann J. C. Weiss J. Lee E. J. Jameson J. L. (2001). Inherited disorders of the gonadotropin hormones. *Mol. Cell. Endocrinol.* 179 89–96 11420133 10.1016/s0303-7207(01)00474-9

[B3] Alarid E. T. Windle J. J. Whyte D. B. Mellon P. L. (1996). Immortalization of pituitary cells at discrete stages of development by directed oncogenesis in transgenic mice. *Development* 122 3319–3329 8898243 10.1242/dev.122.10.3319

[B4] Albarracin C. T. Frosch M. P. Chin W. W. (1999). The gonadotropin-releasing hormone receptor gene promoter directs pituitary-specific oncogene expression in transgenic mice. *Endocrinology* 140 2415–2421 10218996 10.1210/endo.140.5.6682

[B5] Albarracin C. T. Kaiser U. B. Chin W. W. (1994). Isolation and characterization of the 5′-flanking region of the mouse gonadotropin-releasing hormone receptor gene. *Endocrinology* 135 2300–2306 7988412 10.1210/endo.135.6.7988412

[B6] Anderson L. (1996). Intracellular mechanisms triggering gonadotrophin secretion. *Rev. Reprod.* 1 193–202 9414457 10.1530/ror.0.0010193

[B7] Aubert M. L. Begeot M. Winiger B. P. Morel G. Sizonenko P. C. Dubois P. M. (1985). Ontogeny of hypothalamic luteinizing hormone-releasing hormone (GnRH) and pituitary GnRHRs in fetal and neonatal rats. *Endocrinology* 116 1565–1576 2982590 10.1210/endo-116-4-1565

[B8] Bachir L. K. Garrel G. Lozach A. Laverrière J. N. Counis R. (2003). The rat pituitary promoter of the neuronal nitric oxide synthase gene contains an Sp1-, LIM homeodomain-dependent enhancer and a distinct bipartite gonadotropin-releasing hormone-responsive region. *Endocrinology* 144 3995–4007 12933674 10.1210/en.2002-0183

[B9] Bachir L. K. Laverrière J. N. Counis R. (2001). Isolation and characterization of a rat nitric oxide synthase type I gene promoter that confers expression and regulation in pituitary gonadotrope cells. *Endocrinology* 142 4631–4642 11606428 10.1210/endo.142.11.8469

[B10] Ban E. Crumeyrolle-Arias M. Latouche J. Leblanc P. Heurtier J. F. Drieu K. (1990). GnRHRs in rat brain, pituitary and testis; modulation following surgical and gonadotropin-releasing hormone agonist-induced castration. *Mol. Cell. Endocrinol.* 70 99–107 2160386 10.1016/0303-7207(90)90063-e

[B11] Bliss S. P. Miller A. Navratil A. M. Xie J. McDonough S. P. Fisher P. J. (2009). ERK signaling in the pituitary is required for female but not male fertility. *Mol. Endocrinol.* 23 1092–1101 19372235 10.1210/me.2009-0030 PMC2703601

[B12] Bodner M. Castrillo J. L. Theill L. E. Deerinck T. Ellisman M. Karin M. (1988). The pituitary-specific transcription factor GHF-1 is a homeobox-containing protein. *Cell* 55 505–518 2902927 10.1016/0092-8674(88)90037-2

[B13] Botté M. C. Chamagne A. M. Carre M. C. Counis R. Kottler M. L. (1998). Fetal expression of GnRH and Gnrhrs in rat testis and ovary. *J. Endocrinol.* 159 179–189 9795356 10.1677/joe.0.1590179

[B14] Braden T. D. Conn P. M. (1992). Activin-A stimulates the synthesis of gonadotropin-releasing hormone receptors. *Endocrinology* 130 2101–2105 1312442 10.1210/endo.130.4.1312442

[B15] Brown P. McNeilly A. S. (1999). Transcriptional regulation of pituitary gonadotrophin subunit genes. *Rev. Reprod.* 4 117–124 10357099 10.1530/ror.0.0040117

[B16] Cheng C. K. Leung P. C. (2005). Molecular biology of gonadotropin-releasing hormone (GnRH)-I, GnRH-II, and their receptors in humans. *Endocr. Rev.* 26 283–306 15561800 10.1210/er.2003-0039

[B17] Cheng C. K. Chow B. K. Leung P. C. (2003). An activator protein 1-like motif mediates 17β-estradiol repression of gonadotropin-releasing hormone receptor promoter via an estrogen receptor alpha-dependent mechanism in ovarian and breast cancer cells. *Mol. Endocrinol.* 17 2613–2629 12947046 10.1210/me.2003-0217

[B18] Cheng C. K. Yeung C. M. Chow B. K. Leung P. C. (2002a). Characterization of a new upstream GnRH receptor promoter in human ovarian granulosa-luteal cells. *Mol. Endocrinol.* 16 1552–1564 12089350 10.1210/mend.16.7.0869

[B19] Cheng C. K. Yeung C. M. Hoo R. L. Chow B. K. Leung P. C. (2002b). Oct-1 is involved in the transcriptional repression of the gonadotropin-releasing hormone receptor gene. *Endocrinology* 143 4693–4701 12446597 10.1210/en.2002-220576

[B20] Cheng K. W. Leung P. C. (2001). Human gonadotropin-releasing hormone receptor gene transcription: up-regulation by 3′,5′-cyclic adenosine monophosphate/protein kinase A pathway. *Mol. Cell. Endocrinol.* 181 15–26 11476937 10.1016/s0303-7207(01)00480-4

[B21] Cheng K. W. Chow B. K. Leung P. C. (2001a). Functional mapping of a placenta-specific upstream promoter for human gonadotropin-releasing hormone receptor gene. *Endocrinology* 142 1506–1516 11250931 10.1210/endo.142.4.8104

[B22] Cheng K. W. Cheng C. K. Leung P. C. (2001b). Differential role of PR-A and -B isoforms in transcription regulation of human Gnrhr. *Mol. Endocrinol.* 15 2078–2092 11731610 10.1210/mend.15.12.0739

[B23] Cheng K. W. Ngan E. S. Kang S. K. Chow B. K. Leung P. C. (2000). Transcriptional down-regulation of human gonadotropin-releasing hormone (GnRH) receptor gene by GnRH: role of protein kinase C and activating protein 1. *Endocrinology* 141 3611–3622 11014215 10.1210/endo.141.10.7730

[B24] Cherrington B. D. Bailey J. S. Diaz A. L. Mellon P. L. (2008). NeuroD1 and Mash1 temporally regulate GnRH receptor gene expression in immortalized mouse gonadotrope cells. *Mol. Cell. Endocrinol.* 295 106–114 18760324 10.1016/j.mce.2008.07.017 PMC2640340

[B25] Cherrington B. D. Farmerie T. A. Clay C. M. (2006). A specific helical orientation underlies the functional contribution of the activin responsive unit to transcriptional activity of the murine gonadotropin-releasing hormone receptor gene promoter. *Endocrine* 29 425–433 16943581 10.1385/ENDO:29:3:425

[B26] Cherrington B. D. Farmerie T. A. Lents C. A. Cantlon J. D. Roberson M. S. Clay C. M. (2005). Activin responsiveness of the murine gonadotropin-releasing hormone receptor gene is mediated by a composite enhancer containing spatially distinct regulatory elements. *Mol. Endocrinol.* 19 898–912 15637149 10.1210/me.2004-0214

[B27] Chevrier L. Guimiot F de Roux N. (2011). GnRH receptor mutations in isolated gonadotropic deficiency. *Mol. Cell. Endocrinol.* 346 21–28 21645587 10.1016/j.mce.2011.04.018

[B28] Clay C. M. Nelson S. E. Digregorio G. B. Campion C. E. Wiedemann A. L. Nett R. J. (1995). Cell-specific expression of the mouse gonadotropin-releasing hormone (GnRH) receptor gene is conferred by elements residing within 500 bp of proximal 5′ flanking region. *Endocrine* 3 615–622 21153141 10.1007/BF02953028

[B29] Connor E. E. Ashwell M. S. Kappes S. M. Dahl G. E. (1999). Mapping of the bovine growth hormone-releasing hormone receptor (GHRH-R) gene to chromosome 4 by linkage analysis using a novel PCR-RFLP. *J. Anim. Sci.* 77 793–794 10229382 10.2527/1999.773793x

[B30] de Roux N. Milgrom E. (2001). Inherited disorders of GnRH and gonadotropin receptors. *Mol. Cell. Endocrinol.* 179 83–87 11420132 10.1016/s0303-7207(01)00471-3

[B31] de Roux N. Young J. Misrahi M. Genet R. Chanson P. Schaison G. (1997). A family with hypogonadotropic hypogonadism and mutations in the gonadotropin-releasing hormone receptor. *N. Engl. J. Med.* 337 1597–1602 9371856 10.1056/NEJM199711273372205

[B32] Delahaye R. Manna P. R. Berault A. Berreur-Bonnenfant J. Berreur P. Counis R. (1997). Rat gonadotropin-releasing hormone receptor expressed in insect cells induces activation of adenylyl cyclase. *Mol. Cell. Endocrinol.* 135 119–127 9484907 10.1016/s0303-7207(97)00194-9

[B33] Drouin J. Lamolet B. Lamonerie T. Lanctôt C. Tremblay J. J. (1998). The PTX family of homeodomain transcription factors during pituitary developments. *Mol. Cell. Endocrinol.* 140 31–36 9722165 10.1016/s0303-7207(98)00026-4

[B34] Duan W. R. Ito M. Park Y. Maizels E. T. Hunzicker-Dunn M. Jameson J. L. (2002). GnRH regulates early growth response protein 1 transcription through multiple promoter elements. *Mol. Endocrinol.* 16 221–233 11818496 10.1210/mend.16.2.0779

[B35] Duval D. L. Ellsworth B. S. Clay C. M. (1999). Is gonadotrope expression of the gonadotropin releasing hormone receptor gene mediated by autocrine/paracrine stimulation of an activin response element? *Endocrinology* 140 1949–1952 10098536 10.1210/endo.140.4.6780

[B36] Duval D. L. Farris A. R. Quirk C. C. Nett T. M. Hamernik D. L. Clay C. M. (2000). Responsiveness of the ovine gonadotropin-releasing hormone receptor gene to estradiol and gonadotropin-releasing hormone is not detectable *in vitro* but is revealed in transgenic mice. *Endocrinology* 141 1001–1010 10698176 10.1210/endo.141.3.7391

[B37] Duval D. L. Nelson S. E. Clay C. M. (1997a). A binding site for steroidogenic factor-1 is part of a complex enhancer that mediates expression of the murine gonadotropin-releasing hormone receptor gene. *Biol. Reprod.* 56 160–168 9002645 10.1095/biolreprod56.1.160

[B38] Duval D. L. Nelson S. E. Clay C. M. (1997b). The tripartite basal enhancer of the gonadotropin-releasing hormone (GnRH) receptor gene promoter regulates cell-specific expression through a novel GnRHR activating sequence. *Mol. Endocrinol.* 11 1814–1821 9369449 10.1210/mend.11.12.0020

[B39] Ellsworth B. S. Burns A. T. Escudero K. W. Duval D. L. Nelson S. E. Clay C. M. (2003a). The gonadotropin releasing hormone (GnRH) receptor activating sequence (GRAS) is a composite regulatory element that interacts with multiple classes of transcription factors including Smads, AP-1 and a forkhead DNA binding protein. *Mol. Cell. Endocrinol.* 206 93–111 12943993 10.1016/s0303-7207(03)00235-1

[B40] Ellsworth B. S. White B. R. Burns A. T. Cherrington B. D. Otis A. M. Clay C. M. (2003b). c-JunN-terminal kinase activation of activator protein-1 underlies homologous regulation of the gonadotropin-releasing hormone receptor gene in alpha T3-1cells. *Endocrinology* 144 839–849 12586760 10.1210/en.2002-220784

[B41] Ericson J. Norlin S. Jessell T. M. Edlund T. (1998). Integrated FGF and BMP signaling controls the progression of progenitor cell differentiation and the emergence of pattern in the embryonic anterior pituitary. *Development* 125 1005–1015 9463347 10.1242/dev.125.6.1005

[B42] Fan N. C. Jeung E. B. Peng C. Olofsson J. I. Krisinger J. Leung P. C. (1994). The human gonadotropin-releasing hormone (GnRH) receptor gene: cloning, genomic organization and chromosomal assignment. *Mol. Cell. Endocrinol.* 103 R1–R6 7958384 10.1016/0303-7207(94)90087-6

[B43] Fan N. C. Peng C. Krisinger J. Leung P. C. (1995). The human gonadotropin-releasing hormone receptor gene: complete structure including multiple promoters, transcription initiation sites, and polyadenylation signals. *Mol. Cell. Endocrinol.* 107 R1–R8 7768323 10.1016/0303-7207(94)03460-b

[B44] Fernandez-Vazquez G. Kaiser U. B. Albarracin C. T. Chin W. W. (1996). Transcriptional activation of the gonadotropin-releasing hormone receptor gene by activin A. *Mol. Endocrinol.* 10 356–366 8721981 10.1210/mend.10.4.8721981

[B45] Garrel G. Delahaye R. Hemmings B. A. Counis R. (1995). Modulation of regulatory and catalytic subunit levels of cAMP-dependent protein kinase A in anterior pituitary cells in response to direct activation of protein kinases A and C or after GnRH stimulation. *Neuroendocrinology* 62 514–522 8559283 10.1159/000127042

[B46] Garrel G. Lerrant Y. Siriostis C. Berault A. Magre S. Bouchaud C. (1998). Evidence that gonadotropin-releasing hormone stimulates gene expression and levels of active nitric oxide synthase type I in pituitary gonadotrophs, a process altered by desensitization and, indirectly, by gonadal steroids. *Endocrinology* 139 2163–2170 9529006 10.1210/endo.139.4.5890

[B47] Garrel G. McArdle C. A. Hemmings B. A. Counis R. (1997). Gonadotropin-releasing hormone and pituitary adenylate cyclase-activating polypeptide affect levels of cyclic adenosine 3′,5′-monophosphate-dependent protein kinase A (PKA) subunits in the clonal gonadotrope alphaT3-1 cells: evidence for cross-talk between PKA and protein kinase C pathways. *Endocrinology* 138 2259–2266 9165009 10.1210/endo.138.6.5187

[B48] Garrel G. Simon V. Thieulant M. L. Cayla X. Garcia A. Counis R. (2010). Sustained gonadotropin-releasing hormone stimulation mobilizes the cAMP/PKA pathway to induce nitric oxide synthase type 1 expression in rat pituitary cells *in vitro* and *in vivo* at proestrus. *Biol. Reprod.* 82 1170–1179 20181617 10.1095/biolreprod.109.082925

[B49] Granger A. Bleux C. Kottler M. L. Rhodes S. J. Counis R Laverrière J. N. (2006). The LIM-homeodomain proteins Isl-1 and Lhx3 act with steroidogenic factor 1 to enhance gonadotrope-specific activity of the gonadotropin-releasing hormone receptor gene promoter. *Mol. Endocrinol.* 20 2093–2108 16613990 10.1210/me.2005-0184

[B50] Granger A. Ngô-Muller V. Bleux C. Guigon C. Pincas H. Magre S. (2004). The promoter of the rat gonadotropin-releasing hormone receptor gene directs the expression of the human placental alkaline phosphatase reporter gene in gonadotrope cells in the anterior pituitary gland as well as in multiple extrapituitary tissues. *Endocrinology* 145 983–993 14592958 10.1210/en.2003-0881

[B51] Greer E. L. Shi Y. (2012). Histone methylation: a dynamic mark in health, disease and inheritance. *Nat. Rev. Genet.* 13 343–357 22473383 10.1038/nrg3173 PMC4073795

[B52] Hamernik D. L. Clay C. M. Turzillo A. Van Kirk E. A. Moss G. E. (1995). Estradiol increases amounts of messenger ribonucleic acid for gonadotropin-releasing hormone receptors in sheep. *Biol. Reprod.* 53 179–185 7669847 10.1095/biolreprod53.1.179

[B53] Han X. B. Conn P. M. (1999). The role of protein kinases A and C pathways in the regulation of mitogen-activated protein kinase activation in response to gonadotropin-releasing hormone receptor activation. *Endocrinology* 140 2241–2251 10218977 10.1210/endo.140.5.6707

[B54] Hapgood J. P. Sadie H. van Biljon W. Ronacher K. (2005). Regulation of expression of mammalian gonadotrophin-releasing hormone receptor genes. *J. Neuroendocrinol.* 17 619–638 16159375 10.1111/j.1365-2826.2005.01353.x

[B55] Hoo R. L. Ngan E. S. Leung P. C. Chow B. K. (2003). Two Inr elements are important for mediating the activity of the proximal promoter of the human gonadotropin-releasing hormone receptor gene. *Endocrinology* 144 518–527 12538612 10.1210/en.2002-220591

[B56] Ikeda Y. Luo X. Abbud R. Nilson J. H. Parker K. L. (1995). The nuclear receptor steroidogenic factor 1 is essential for the formation of the ventromedial hypothalamic nucleus. *Mol. Endocrinol.* 9 478–486 7659091 10.1210/mend.9.4.7659091

[B57] Ingraham H. A. Chen R. P. Mangalam H. J. Elsholtz H. P. Flynn S. E. Lin C. R. (1988). A tissue-specific transcription factor containing a homeodomain specifies a pituitary phenotype. *Cell* 55 519–529 2902928 10.1016/0092-8674(88)90038-4

[B58] Ingraham H. A. Lala D. S. Ikeda Y. Luo X. Shen W. H. Nachtigal M. W. (1994). The nuclear receptor steroidogenic factor 1 acts at multiple levels of the reproductive axis. *Genes Dev.* 8 2302–2312 7958897 10.1101/gad.8.19.2302

[B59] Ito M. Park Y. Weck J. Mayo K. E. Jameson J. L. (2000). Synergistic activation of the inhibin alpha-promoter by steroidogenic factor-1 and cyclic adenosine 3′,5′-monophosphate. *Mol. Endocrinol.* 14 66–81 10628748 10.1210/mend.14.1.0410

[B60] Janovick J. A. Conn P. M. (1994). Gonadotropin-releasing hormone (GnRH)-receptor coupling to inositol phosphate and prolactin production in GH3 cells stably transfected with rat GnRHR complementary deoxyribonucleic acid. *Endocrinology* 135 2214–2219 7956944 10.1210/endo.135.5.7956944

[B61] Japón M.A. Rubinstein M. Low M. J. (1994). *In situ* hybridization analysis of anterior pituitary hormone gene expression during fetal mouse development. *J. Histochem. Cytochem*. 42 1117–1125 8027530 10.1177/42.8.8027530

[B62] Jennes L. (1990). Prenatal development of gonadotropin-releasing hormone receptors in the rat anterior pituitary. *Endocrinology* 126 942–947 2153535 10.1210/endo-126-2-942

[B63] Jennes L. Brame B. Centers A. Janovick J. A. Conn P. M. (1995). Regulation of hippocampal gonadotropin releasing hormone (GnRH) receptor mRNA and GnRH-stimulated inositol phosphate production by gonadal steroid hormones. *Brain Res. Mol. Brain Res.* 33 104–110 8774951 10.1016/0169-328x(95)00113-7

[B64] Jennes L. Dalati B. Conn P. M. (1988). Distribution of gonadrotropin releasing hormone agonist binding sites in the rat central nervous system. *Brain Res.* 452 156–164 2841008 10.1016/0006-8993(88)90020-0

[B65] Jennes L. Eyigor O. Janovick J. A. Conn P. M. (1997). Brain gonadotropin releasing hormone receptors: localization and regulation. *Recent Prog. Horm. Res.* 52 475–490 9238864

[B66] Jeong K. H. Chin W. W. Kaiser U. B. (2004). Essential role of the homeodomain for pituitary homeobox 1 activation of mouse gonadotropin-releasing hormone receptor gene expression through interactions with c-Jun and DNA. *Mol. Cell. Biol.* 24 6127–6139 15226417 10.1128/MCB.24.14.6127-6139.2004 PMC434250

[B67] Jeong K. H. Gill J. C. Nosé V. Parlow A. F. Carroll R. S. Kaiser U. B. (2009). Expression of a gonadotropin-releasing hormone receptor-simian virus 40 T-antigen transgene has sex-specific effects on the reproductive axis. *Endocrinology* 150 3383–3391 19282386 10.1210/en.2008-1362 PMC2703545

[B68] Jiang Z. Gibson J. P. Archibald A. L. Haley C. S. (2001). The porcine gonadotropin-releasing hormone receptor gene (GNRHR): genomic organization, polymorphisms, and association with the number of corpora lutea. *Genome* 44 7–12 11269358 10.1139/gen-44-1-7

[B69] Kaiser U. B. Dushkin H. Altherr M. R. Beier D. R. Chin W. W. (1994). Chromosomal localization of the gonadotropin-releasing hormone receptor gene to human chromosome 4q13.1-q21.1 and mouse chromosome 5. *Genomics* 20 506–508 8034328 10.1006/geno.1994.1211

[B70] Kaiser U. B. Katzenellenbogen R. A. Conn P. M. Chin W. W. (1994). Evidence that signalling pathways by which thyrotropin-releasing hormone and gonadotropin-releasing hormone act are both common and distinct. *Mol. Endocrinol.* 8 1038–1048 7527898 10.1210/mend.8.8.7527898

[B71] Kakar S. S. (1997). Molecular structure of the human gonadotropin-releasing hormone receptor gene. *Eur. J. Endocrinol.* 137 183–192 9272108 10.1530/eje.0.1370183

[B72] Kakar S. S. Neill J. D. (1995). The human gonadotropin-releasing hormone receptor gene (GNRHR) maps to chromosome band 4q13. *Cytogenet. Cell Genet.* 70 211–214 7789173 10.1159/000134035

[B73] Kakar S. S. Winters S. J. Zacharias W. Miller D. M. Flynn S. (2003). Identification of distinct gene expression profiles associated with treatment of LbetaT2 cells with gonadotropin-releasing hormone agonist using microarray analysis. *Gene* 308 67–77 12711391 10.1016/s0378-1119(03)00446-3

[B74] Kam K. Y. Jeong K. H. Norwitz E. R. Jorgensen E. M. Kaiser U. B. (2005). Oct-1 and nuclear factor Y bind to the SURG-1 element to direct basal and gonadotropin-releasing hormone (GnRH)-stimulated mouse GnRH receptor gene transcription. *Mol. Endocrinol.* 19 148–162 15388790 10.1210/me.2004-0025

[B75] Kang S. K. Cheng K. W. Ngan E. S. Chow B. K. Choi K. C. Leung P. C. (2000). Differential expression of human gonadotropin-releasing hormone receptor gene in pituitary and ovarian cells. *Mol. Cell. Endocrinol.* 162 157–166 10854709 10.1016/s0303-7207(00)00196-9

[B76] Kappen C. Salbaum J. M. (2009). Identification of regulatory elements in the Isl1 gene locus. *Int. J. Dev. Biol.* 53 935–946 19598113 10.1387/ijdb.082819ck PMC3482124

[B77] Kawaminami M. Etoh S. Miyaoka H. Sakai M. Nishida M. Kurusu S. (2002). Annexin 5 messenger ribonucleic acid expression in pituitary gonadotropes is induced by gonadotropin-releasing hormone (GnRH) and modulates GnRH stimulation of gonadotropin release. *Neuroendocrinology* 75 2–11 11810030 10.1159/000048216

[B78] Kirkpatrick B. L. Esquivel E. Moss G. E. Hamernik D. L. Wise M. E. (1998). Estradiol and gonadotropin-releasing hormone (GnRH) interact to increase GnRH receptor expression in ovariectomized ewes after hypothalamic-pituitary disconnection. *Endocrine* 8 225–229 9741826 10.1385/ENDO:8:3:225

[B79] Kottler M. L. Lorenzo F. Bergametti F. Commercon P. Souchier C. Counis R. (1995). Subregional mapping of the human gonadotropin-releasing hormone receptor (GnRHR) gene to 4q between the markers D4S392 and D4S409. *Hum. Genet.* 96 477–480 7557974 10.1007/BF00191810

[B80] Kraus S. Naor Z. Seger R. (2001). Intracellular signaling pathways mediated by the gonadotropin-releasing hormone (GnRH) receptor. *Arch. Med. Res.* 32 499–509 11750725 10.1016/s0188-4409(01)00331-9

[B81] Ku M. Koche R. P. Rheinbay E. Mendenhall E. M. Endoh M. Mikkelsen T. S. (2008). Genomewide analysis of PRC1 and PRC2 occupancy identifies two classes of bivalent domains. *PLoS Genet. *4:e1000242. 10.1371/journal.pgen.1000242 PMC2567431 18974828

[B82] Larivière S. Garrel G. Simon V. Soh J. W. Laverrière J. N. Counis R. (2007). Gonadotropin-releasing hormone couples to 3′,5′-cyclic adenosine-5′-monophosphate pathway through novel protein kinase Cδ and -ε in LβT2 gonadotrope cells. *Endocrinology* 148 1099–1107 17185372 10.1210/en.2006-1473

[B83] Larivière S. Garrel-Lazayres G. Simon V. Shintani N. Baba A. Counis R. (2008). Gonadotropin-releasing hormone inhibits pituitary adenylyl cyclase-activating polypeptide coupling to 3′,5′-cyclic adenosine-5′-monophosphate pathway in LβT2 gonadotrope cells through novel protein kinase C isoforms and phosphorylation of pituitary adenylyl cyclase-activating polypeptide type I receptor. *Endocrinology* 149 6389–6398 18755795 10.1210/en.2008-0504

[B84] Layman L. C. Cohen D. P. Jin M. Xie J. Li Z. Reindollar R. H. (1998). Mutations in gonadotropin-releasing hormone receptor gene cause hypogonadotropic hypogonadism. *Nat. Genet.* 18 14–15 9425890 10.1038/ng0198-14

[B85] Leblanc P. Crumeyrolle M. Latouche J. Jordan D. Fillion G. L’Heritier A. (1988). Characterization and distribution of receptors for gonadotropin-releasing hormone in the rat hippocampus. *Neuroendocrinology* 48 482–488 2854220 10.1159/000125053

[B86] Lents C. A. Farmerie T. A. Cherrington B. D. Clay C. M. (2009). Multiple core homeodomain binding motifs differentially contribute to transcriptional activity of the murine gonadotropin-releasing hormone receptor gene promoter. *Endocrine* 35 356–364 19333792 10.1007/s12020-009-9167-1

[B87] Leung P. C. Squire J. Peng C. Fan N. Hayden M. R. Olofsson J. I. (1995). Mapping of the gonadotropin-releasing hormone (GnRH) receptor gene to human chromosome 4q21.2 by fluorescence in situ hybridization. *Mamm. Genome* 6 309–310 7613048 10.1007/BF00352431

[B88] Li J. Liu Q. Qiu M. Pan Y. Li Y. Shi T. (2006). Identification and analysis of the mouse basic/helix-loop-helix transcription factor family. *Biochem. Biophys. Res. Commun.* 350 648–656 17027923 10.1016/j.bbrc.2006.09.114

[B89] Li S. Crenshaw E. B. III Rawson E. J. Simmons D. M. Swanson L. W. Rosenfeld M. G. (1990). Dwarf locus mutants lacking three pituitary cell types result from mutations in the POU-domain gene Pit-1. *Nature* 347 528–533 1977085 10.1038/347528a0

[B90] Lienert F. Wirbelauer C. Som I. Dean A. Mohn F. Schübeler D. (2011). Identification of genetic elements that autonomously determine DNA methylation states. *Nat. Genet.* 43 1091–1097 21964573 10.1038/ng.946

[B91] Lin X. Conn P. M. (1998). Transcriptional activation of gonadotropin-releasing hormone (GnRH) receptor gene by GnRH and cyclic adenosine monophosphate. *Endocrinology* 139 3896–3902 9724045 10.1210/endo.139.9.6214

[B92] Lin X. Conn P. M. (1999). Transcriptional activation of gonadotropin-releasing hormone (GnRH) receptor gene by GnRH: involvement of multiple signal transduction pathways. *Endocrinology* 140 358–364 9886846 10.1210/endo.140.1.6452

[B93] Liu F. Austin D. A. Mellon P. L. Olefsky J. M. Webster N. J. (2002). GnRH activates ERK1/2 leading to the induction of c-fos and LHβ protein expression in LβT2 cells. *Mol. Endocrinol.* 16 419–434 11875099 10.1210/mend.16.3.0791

[B94] Lozach A. Garrel G. Lerrant Y. Berault A. Counis R. (1998). GnRH-dependent up-regulation of nitric oxide synthase I level in pituitary gonadotrophs mediates cGMP elevation during rat proestrus. *Mol. Cell. Endocrinol.* 143 43–51 9806349 10.1016/s0303-7207(98)00135-x

[B95] Luo X. Ikeda Y. Schlosser D. A. Parker K. L. (1995). Steroidogenic factor 1 is the essential transcript of the mouse Ftz-F1 gene. *Mol. Endocrinol.* 9 1233–1239 7491115 10.1210/mend.9.9.7491115

[B96] Maya-Nunez G. Conn P. M. (1999). Transcriptional regulation of the gonadotropin-releasing hormone receptor gene is mediated in part by a putative repressor element and by the cyclic adenosine 3′,5′-monophosphate response element. *Endocrinology* 140 3452–3458 10433200 10.1210/endo.140.8.6945

[B97] Maya-Nunez G. Conn P. M. (2001). Cyclic adenosine 3′,5′-monophosphate (cAMP) and cAMP responsive element-binding protein are involved in the transcriptional regulation of gonadotropin-releasing hormone (GnRH) receptor by GnRH and mitogen-activated protein kinase signal transduction pathway in GGH(3) cells. *Biol. Reprod.* 65 561–567 11466226 10.1095/biolreprod65.2.561

[B98] McCue J. M. Quirk C. C. Nelson S. E. Bowen R. A. Clay C. M. (1997). Expression of a murine gonadotropin-releasing hormone receptor-luciferase fusion gene in transgenic mice is diminished by immunoneutralization of gonadotropin-releasing hormone. *Endocrinology* 138 3154–3160 9231762 10.1210/endo.138.8.5306

[B99] McGillivray S. M. Bailey J. S. Ramezani R. Kirkwood B. J. Mellon P. L. (2005). Mouse GnRH receptor gene expression is mediated by the LHX3 homeodomain protein. *Endocrinology* 146 2180–2185 15705775 10.1210/en.2004-1566 PMC2930620

[B100] Meldi L. Brickner J. H. (2011). Compartmentalization of the nucleus. *Trends Cell Biol.* 21 701–708 21900010 10.1016/j.tcb.2011.08.001 PMC3970429

[B101] Montgomery G. W. Penty J. M. Lord E. A. Brooks J. McNeilly A. S. (1995). The gonadotrophin-releasing hormone receptor maps to sheep chromosome 6 outside of the region of the FecB locus. *Mamm. Genome* 6 436–438 7647469 10.1007/BF00355648

[B102] Morrison N. Sellar R. E. Boyd E. Eidne K. A. Connor J. M. (1994). Assignment of the gene encoding the human gonadotropin-releasing hormone receptor to 4q13.2-13.3 by fluorescence in situ hybridization. *Hum. Genet.* 93 714–715 8005601 10.1007/BF00201579

[B103] Mullen R. D. Park S. Rhodes S. J. (2012). A distal modular enhancer complex acts to control pituitary- and nervous system-specific expression of the LHX3 regulatory gene. *Mol. Endocrinol.* 26 308–319 22194342 10.1210/me.2011-1252 PMC3275162

[B104] Ngan E. S. Cheng P. K. Leung P. C. Chow B. K. (1999). Steroidogenic factor-1 interacts with a gonadotrope-specific element within the first exon of the human gonadotropin-releasing hormone receptor gene to mediate gonadotrope-specific expression. *Endocrinology* 140 2452–2462 10342829 10.1210/endo.140.6.6759

[B105] Ngan E. S. Leung P. C. Chow B. K. (2000). Identification of an upstream promoter in the human gonadotropin-releasing hormone receptor gene. *Biochem. Biophys. Res. Commun.* 270 766–772 10772899 10.1006/bbrc.2000.2509

[B106] Ngan E. S. Leung P. C. Chow B. K. (2001). Interplay of pituitary adenylate cyclase-activating polypeptide with a silencer element to regulate the upstream promoter of the human gonadotropin-releasing hormone receptor gene. *Mol. Cell. Endocrinol.* 176 135–144 11369453 10.1016/s0303-7207(01)00402-6

[B107] Ngô V. M. Laverrière J. N. Gourdji D. (1995). CpG methylation represses the activity of the rat prolactin promoter in rat GH3 pituitary cell lines. *Mol. Cell. Endocrinol.* 108 95–105 7538957 10.1016/0303-7207(94)03462-3

[B108] Noel S. D. Kaiser U. B. (2011). G protein-coupled receptors involved in GnRH regulation: molecular insights from human disease. *Mol. Cell. Endocrinol.* 346 91–101 21736917 10.1016/j.mce.2011.06.022 PMC3185177

[B109] Norwitz E. R. Cardona G. R. Jeong K. H. Chin W. W. (1999b). Identification and characterization of the gonadotropin-releasing hormone response elements in the mouse gonadotropin-releasing hormone receptor gene. *J. Biol. Chem.* 274 867–880 9873026 10.1074/jbc.274.2.867

[B110] Norwitz E. R. Jeong K. H. Chin W. W. (1999a). Molecular mechanisms of gonadotropin-releasing hormone receptor gene regulation. *J. Soc. Gynecol. Investig.* 6 169–178 10.1016/s1071-5576(99)00022-2 10486777

[B111] Norwitz E. R. Xu S. Jeong K. H. Bedecarrats G. Y. Winebrenner L. D. Chin W. W. (2002a). Activin A augments GnRH-mediated transcriptional activation of the mouse Gnrhr. *Endocrinology* 143 985–997 11861523 10.1210/endo.143.3.8663

[B112] Norwitz E. R. Xu S. Xu J. Spiryda L. B. Park J. S. Jeong K. H. (2002b). Direct binding of AP-1 (Fos/Jun) proteins to a SMAD binding element facilitates both gonadotropin-releasing hormone (GnRH)- and activin-mediated transcriptional activation of the mouse GnRH receptor gene. *J. Biol. Chem.* 277 37469–37478 12145309 10.1074/jbc.M206571200

[B113] Pincas H. Amoyel K. Counis R. Laverriere J. N. (2001a). Proximal cis-acting elements, including steroidogenic factor 1, mediate the efficiency of a distal enhancer in the promoter of the rat gonadotropin-releasing hormone receptor gene. *Mol. Endocrinol.* 15 319–337 11158337 10.1210/mend.15.2.0593

[B114] Pincas H. Laverriere J. N. Counis R. (2001b). Pituitary adenylate cyclase-activating polypeptide and cyclic adenosine 3′,5′-monophosphate stimulate the promoter activity of the rat gonadotropin-releasing hormone receptor gene via a bipartite response element in gonadotrope-derived cells. *J. Biol. Chem.* 276 23562–23571 11320087 10.1074/jbc.M100563200

[B115] Pincas H. Forrai Z. Chauvin S. Laverriere J. N. Counis R. (1998). Multiple elements in the distal part of the 1.2 kb 5′-flanking region of the rat Gnrhr regulate gonadotrope-specific expression conferred by proximal domain. *Mol. Cell. Endocrinol.* 144 95–108 9863630 10.1016/s0303-7207(98)00149-x

[B116] Pirrotta V. Li H. B. (2012). A view of nuclear polycomb bodies. *Curr. Opin. Genet. Dev.* 22 101–109 22178420 10.1016/j.gde.2011.11.004 PMC3329586

[B117] Reinhart J. Xiao S. Arora K. K. Catt K. J. (1997). Structural organization and characterization of the promoter region of the rat gonadotropin-releasing hormone receptor gene. *Mol. Cell. Endocrinol.* 130 1–12 9220016 10.1016/s0303-7207(97)00064-6

[B118] Resuehr D. Wildemann U. Sikes H. Olcese J. (2007). E-box regulation of gonadotropin-releasing hormone (GnRH) receptor expression in immortalized gonadotrope cells. *Mol. Cell. Endocrinol.* 278 36–43 17928134 10.1016/j.mce.2007.08.008

[B119] Roesler W. J. (2000). What is a cAMP response unit? *Mol. Cell. Endocrinol.* 162 1–7 10854692 10.1016/s0303-7207(00)00198-2

[B120] Sadie H. Styger G. Hapgood J. (2003). Expression of the mouse gonadotropin-releasing hormone receptor gene in alpha T3-1 gonadotrope cells is stimulated by cyclic 3′,5′-adenosine monophosphate and protein kinase A, and is modulated by steroidogenic factor-1 and Nur77. *Endocrinology.* 144 1958–1971 12697703 10.1210/en.2002-220874

[B121] Schang A. L. Bleux C. Chenut M. C. Ngô-Muller V. Quérat B. Jeanny J. C. (2012a). Identification and analysis of two novel sites of rat GnRH receptor gene promoter activity: the pineal gland and retina. *Neuroendocrinology*. 10.1159/000337661 [Epub ahead of print]. 22414758

[B122] Schang A. L. Granger A. Quérat B. Bleux C. Cohen-Tannoudji J. Laverrière J. N. (2012b). GATA2-induced silencing and LIM-homeodomain protein-induced activation are mediated by a bi-functional response element in the rat GnRH receptor gene. *Mol. Endocrinol.* (in press) 10.1210/me.2012-1182 PMC5416942 23211524

[B123] Schang A. L. Counis R. Magre S. Bleux C. Granger A. Ngô-Muller V. (2011a). Reporter transgenic mouse models highlight the dual endocrine and neural facet of GnRH receptor function. *Ann. N. Y. Acad. Sci.* 1220 16–22 21388400 10.1111/j.1749-6632.2010.05886.x

[B124] Schang A. L. Ngô-Muller V. Bleux C. Granger A. Chenut M. C. Loudes C. (2011b). GnRH receptor gene expression in the developing rat hippocampus: transcriptional regulation and potential roles in neuronal plasticity. *Endocrinology* 152 568–580 21123436 10.1210/en.2010-0840

[B125] Schlosser G. (2006). Induction and specification of cranial placodes. *Dev. Biol.* 294 303–351 16677629 10.1016/j.ydbio.2006.03.009

[B126] Schuettengruber B. Chourrout D. Vervoort M. Leblanc B. Cavalli G. (2007). Genome regulation by polycomb and trithorax proteins. *Cell* 128 735–745 17320510 10.1016/j.cell.2007.02.009

[B127] Scully K. M. Rosenfeld M. G. (2002). Pituitary development: regulatory codes in mammalian organogenesis. *Science* 295 2231–2235 11910101 10.1126/science.1062736

[B128] Shima Y. Miyabayashi K. Baba T. Otake H. Oka S. Zubair M. (2012). Identification of an enhancer in the Ad4BP/SF-1 gene specific for fetal Leydig cells. *Endocrinology* 153 417–425 22128023 10.1210/en.2011-1407

[B129] Silveira L. F. Stewart P. M. Thomas M. Clark D. A. Bouloux P. M. MacColl G. S. (2002). Novel homozygous splice acceptor site GnRHR (GnRHR) mutation: human GnRHR “knockout.” *J. Clin. Endocrinol. Metab.* 87 2973–2977 12050282 10.1210/jcem.87.6.8535

[B130] Simon J. A. Kingston R. E. (2009). Mechanisms of polycomb gene silencing: knowns and unknowns. *Nat. Rev. Mol. Cell Biol.* 10 697–708 19738629 10.1038/nrm2763

[B131] Sosnowski R. Mellon P. L. Lawson M. A. (2000). Activation of translation in pituitary gonadotrope cells by gonadotropin-releasing hormone. *Mol. Endocrinol.* 14 1811–1819 11075814 10.1210/mend.14.11.0550

[B132] Spector D. L. Lamond A. I. (2011). Nuclear speckles. *Cold Spring Harb. Perspect. Biol.* 3 a000646 10.1101/cshperspect.a000646 PMC3039535 20926517

[B133] Stanislaus D. Arora V. Awara W. M. Conn P. M. (1996). Biphasic action of cyclic adenosine 3′,5′- monophosphate in gonadotropin-releasing hormone (GnRH) analog-stimulated hormone release from GH3 cells stably transfected with GnRHR complementary deoxyribonucleic acid. *Endocrinology* 137 1025–1031 8603570 10.1210/endo.137.3.8603570

[B134] Stanislaus D. Janovick J. A. Jennes L. Kaiser U. B. Chin W. W. Conn P. M. (1994). Functional and morphological characterization of four cell lines derived from GH3 cells stably transfected with gonadotropin-releasing hormone receptor complementary deoxyribonucleic acid. *Endocrinology* 135 2220–2227 7956945 10.1210/endo.135.5.7956945

[B135] Stewart A. J. Katz A. A. Millar R. P. Morgan K. (2009). Retention and silencing of prepro-GnRH-II and type II GnRH receptor genes in mammals. *Neuroendocrinology* 90 416–432 19657181 10.1159/000233303

[B136] Stojilkovic S. S. Reinhart J. Catt K. J. (1994). Gonadotropin-releasing hormone receptors: structure and signal transduction pathways. *Endocr. Rev.* 15 462–499 7988482 10.1210/edrv-15-4-462

[B137] Stopa M. Anhuf D. Terstegen L. Gatsios P. Gressner A. M. Dooley S. (2000). Participation of Smad2, Smad3, and Smad4 in transforming growth factor beta (TGF-beta)-induced activation of Smad7. The TGF-beta response element of the promoter requires functional Smad binding element and E-box sequences for transcriptional regulation. *J. Biol. Chem.* 275 29308–29317 10887185 10.1074/jbc.M003282200

[B138] Thomas P. Mellon P. L. Turgeon J. Waring D. W. (1996). The L beta T2 clonal gonadotrope: a model for single cell studies of endocrine cell secretion. *Endocrinology* 137 2979–2989 8770922 10.1210/endo.137.7.8770922

[B139] Turgeon J. L. Kimura Y. Waring D. W. Mellon P. L. (1996). Steroid and pulsatile gonadotropin-releasing hormone (GnRH) regulation of luteinizing hormone and GnRH receptor in a novel gonadotrope cell line. *Mol. Endocrinol.* 10 439–450 8721988 10.1210/mend.10.4.8721988

[B140] Turzillo A. M. DiGregorio G. B. Nett T. M. (1995). Messenger ribonucleic acid for gonadotropin-releasing hormone receptor and numbers of gonadotropin releasing hormone receptors in ovariectomized ewes after hypothalamic pituitary disconnection and treatment with estradiol. *J. Anim. Sci.* 73 1784–1788 7673072 10.2527/1995.7361784x

[B141] Vazquez-Martinez R. Shorte S. L. Boockfor F. R. Frawley L. S. (2001). Synchronized exocytotic bursts from gonadotropin-releasing hormone-expressing cells: dual control by intrinsic cellular pulsatility and gap junctional communication. *Endocrinology* 142 2095–2101 11316777 10.1210/endo.142.5.8123

[B142] Walton K. L. Makanji Y. Harrison C. A. (2012). New insights into the mechanisms of activin action and inhibition. *Mol. Cell. Endocrinol.* 359 2–12 21763751 10.1016/j.mce.2011.06.030

[B143] Weiss J. Crowley W. F. Jr Halvorson L. M. Jameson J. L. (1993). Perifusion of rat pituitary cells with gonadotropin-releasing hormone, activin, and inhibin reveals distinct effects on gonadotropin gene expression and secretion. *Endocrinology* 132 2307–2311 8504735 10.1210/endo.132.6.8504735

[B144] Wen S. Schwarz J. R. Niculescu D. Dinu C. Bauer C. K. Hirdes W. (2008). Functional characterization of genetically labeled gonadotropes. *Endocrinology* 149 2701–2711 18325995 10.1210/en.2007-1502

[B145] White B. R. Duval D. L. Mulvaney J. M. Roberson M. S. Clay C. M. (1999). Homologous regulation of the gonadotropin-releasing hormone receptor gene is partially mediated by protein kinase C activation of an activator protein-1 element. *Mol. Endocrinol.* 13 566–577 10194763 10.1210/mend.13.4.0262

[B146] Wilson M. D. Barbosa-Morais N. L. Schmidt D. Conboy C. M. Vanes L. Tybulewicz V. L. (2008). Species-specific transcription in mice carrying human chromosome 21. *Science* 322 434–438 18787134 10.1126/science.1160930 PMC3717767

[B147] Windle J. J. Weiner R. I. Mellon P L. (1990). Cell lines of the pituitary gonadotrope lineage derived by targeted oncogenesis in transgenic mice. *Mol. Endocrinol.* 4 597–603 1704103 10.1210/mend-4-4-597

[B148] Yang L. Lin C. Liu W. Zhang J. Ohgi K. A. Grinstein J. D. (2011). ncRNA- and Pc2 methylation-dependent gene relocation between nuclear structures mediates gene activation programs. *Cell* 147 773–788 22078878 10.1016/j.cell.2011.08.054 PMC3297197

[B149] Yeung C. M. An B. S. Cheng C. K. Chow B. K. Leung P. C. (2005). Expression and transcriptional regulation of the GnRH receptor gene in human neuronal cells. *Mol. Hum. Reprod.* 11 837–842 16364974 10.1093/molehr/gah241

[B150] Zhao L. Bakke M. Parker K. L. (2001). Pituitary-specific knockout of steroidogenic factor 1. *Mol. Cell. Endocrinol.* 185 27–32 11738791 10.1016/s0303-7207(01)00621-9

